# Exploring Health Systems Within the Context of Social Determinants of Health: A Global Health Case Study

**DOI:** 10.15766/mep_2374-8265.10457

**Published:** 2016-09-23

**Authors:** Norman B. Fredrick

**Affiliations:** 1 Assistant Professor, Department of Family and Community Medicine, Pennsylvania State University College of Medicine

**Keywords:** Social Determinants of Health, Global Health, Health Systems, Health Care Systems

## Abstract

**Introduction:**

Global health case studies can highlight, often in dramatic fashion, topics such as health disparities, health systems, and the social determinants of health. This resource presents a case study of an adolescent Haitian boy requiring lifesaving heart surgery in the US due to an acquired mitral valve disease from untreated streptococcal pharyngitis.

**Methods:**

While the case begins in a US clinic setting, it then goes on to explore the patient's home context—an impoverished village in rural Haiti—in order to demonstrate the effect of social determinants on both an individual's health and the capacity of health care systems to provide appropriate care. The case study concludes by asking the students to explore which health interventions, from a list of options, they would recommend to improve health in rural Haiti. Students discuss the advantages and disadvantages of developing health initiatives that vary by level of impact and approach.

**Results:**

This case study has been presented to two classes of first-year medical students during their Health Systems course and was well received.

**Discussion:**

By providing linkages between the local and international contexts, this case provides greater relevance for global health material to all participating students.

## Educational Objectives

By the end of the session, students will be able to:

1. Demonstrate an awareness of the larger social and economic context in which health systems exist.
2. Recognize the importance of viewing an individual's health within the context of community resources, including the availability of health systems.
3. Discuss how a patient's capacity to pursue health care services is affected by the larger social and economic context.

## Introduction

An individual's health and ability to seek health care services are affected by a complex interplay of social, economic, and environmental factors within a community. Health systems are defined as “all organizations, people and actions whose primary interest is to promote, restore or maintain health.”^1^ Health systems are a community resource for health, and the availability of even basic health systems can have significant impact on individual and population health.

The contributions of the social determinants of health at both the individual and population levels are most apparent in the setting of extremely impoverished communities. This is often best expressed by demonstrating the effect that one's birthplace has on longevity, and average life expectancy at birth can consequently vary by as many as 40 years.^2^ As described by the Commission on the Education of Health Professionals for the 21st Century, “mainstreaming a comparative global perspective can enrich existing curricula, thereby reducing the demand for extra space and time.”^3^ While the field of global health is rich with examples from which to draw, challenges exist in translating those valuable lessons so that all students perceive relevance of the material to their future clinical practice. This module was developed to introduce important global health perspectives into my institution's first-year medical school curriculum.

The case-based approach was used to engage learners with a plausible encounter in their clinical practice. In developing the case, the learning themes (e.g., social determinants, health system components) were identified first, and then the case was written in a US setting with a plausible scenario. By placing the case in the setting of a US clinic, the presenting problem becomes relevant for all learners regardless of their personal interest in global health. As the case unfolds, learners are introduced to life in rural Haiti and invited to explore a world where there is no basic health care in place. Stripped of almost all the usual health care resources, students then have to decide from a short list of options which health intervention they believe most likely to succeed if implemented given the social-ecological context and the health impact pyramid.^4^ Students are instructed to evaluate each of the proposed health interventions and should see how they correlate from a health systems perspective: One health intervention (build a clinic) focuses on the top of the health impact pyramid, another option (child health campaigns) moves down the pyramid to the community level, and the final option (well drilling) moves even further down the pyramid by attending to the environmental determinants of health.

All students can contribute to the discussion by bringing in their personal experiences and prior knowledge. Those students with prior global health experience may provide important perspectives, but the case is designed to generate critical thinking by students who have no experiences in international or underserved settings. This is also true of the small-group facilitators who will guide the discussion. The case encourages critical and systems thinking. Through this process, the students learn about health system components and explore both how the components interact and interrelate and how health systems are affected by the social determinants of health.

This case adds to the growing set of educational resources in MedEdPORTAL aimed at providing instructors with accessible content related to both global health and the social determinants of health. MedEdPORTAL is ideally suited to attract these important curricular opportunities and has published a number of resources relevant to global health and the social determinants, including those on refugee health,^5^ homelessness,^6^ health care disparities,^7^ equity,^8^ the One Health initiative,^9^ global health ethics,^10^ pandemic flu,^11^ and various clinical topics.^12^ While this wide variety of excellent global health case studies is useful, far less available are case studies that apply to all students regardless of their geographical scope of interest. There is ongoing debate about the way in which global health competencies should be implemented, both for those interested in global health^13^ and for all students,^14^ but this case study attempts to bridge that gap. By using the global health context as a means of teaching important medical education topics that align with Liaison Committee on Medical Education (LCME) standards (i.e., social sciences [7.1], impact of behavioral and social factors [7.2], societal problems [7.5], and health care disparities [7.6]^15^), the content becomes appropriate for all medical students.

## Methods

This 2-hour session centers around a real case written from personal experiences in rural Haiti, though the names of people and places have been changed. In my medical school's curriculum, the case is taught during the first-year Health Systems course, which coincides with the students’ cardiology block. The case, which features an adolescent male with a heart problem, integrates material between the two courses and is therefore ideally suited for medical students who have some knowledge of cardiology. However, cardiology knowledge is not essential as the medical mystery aspect of the case only serves to open up a discussion about a health condition rarely seen in the US and to emphasize the impact that poverty has on health. The case could also be implemented with residents and, with some modification, could be adapted for public health students and other health professional students.

Each small group is facilitated by a faculty member so the total number of facilitators depends on the number of student groups. The materials are provided to the facilitators 1 week in advance for review. Prior knowledge of the particular case or of global health issues is not necessary for facilitators. Students receive their materials during the small-group session. At my institution, there is no required prereading because of students’ prior work in the Health Systems course; however, useful prereadings may include (1) the Overview of the World Health Systems Framework site,^1^ which provides a brief overview of the six building blocks of health systems that in turn interact to affect both access to and quality of care, and (2) an article by Mills^16^ that provides an overview of common constraints on health care systems and strategies to strengthen them.

The session begins with a 50-minute small-group problem-based learning session. Students are assigned to small groups of eight with a facilitator to work through the scenario. The facilitator version of the case (Appendix A) includes additional notes in italics as well as instructions on when to hand out each of the three parts of the student version of the case (Appendix B). It is useful for small-group facilitators to designate a timekeeper in order to keep the group on schedule. The small-group session concludes with the students coming to a consensus as to which health intervention they will vote for in the large-group session that follows.

For the large-group session, students should sit with their small groups in order to continue to deliberate over the material and vote at different points in the presentation (Appendix C). The large-group session begins with each group voting (via voting cards or an audience response system) on its proposed health intervention and justifying that choice to the class. After each group presents its rationale, additional information is provided to the groups in a packet (Appendix E). The small groups reconvene and examine the new information for 10 minutes. They are then called on to vote again and describe whether the new information caused them to change their vote or not. After a brief discussion, the facilitator then describes the effects of each of the four interventions after real-world application. Details of the presentation preparation and speaker's notes are included in Appendix D.

Successful implementation of this session requires small-group rooms, the ability to project a PowerPoint presentation, an audience response system or four voting cards (labeled A, B, C, and D), and a large-group room set up to allow students to sit in their small groups.

## Results

This session was presented to 145 first-year medical students during the 2015–2016 academic year. Student and faculty evaluations were very positive. Students indicated that the lecture supported their mastery of the learning objectives (*M* = 4.1, 5-point Likert scale). The learning resources were similarly rated (*M* = 4.2, 5-point Likert scale). The Health System course directors received very positive feedback from the students: “At the Just in Time feedback session yesterday, the students raved about Ben Fredrick's session.” Other student comments included the following:

• “I think this was one of the best lectures we have had. Not only was it very interesting, it was also very applicable. Cool to see how far a dollar can go in these developing nations.”
• “This was one of the best lectures we have had in Health Systems.”
• “Made me think a lot, both during the session and afterwards. Provoked some good discussions with friends afterward, for example.”
• “This Sync session has been one of the more interesting sessions because it has truly highlighted how medicine works in many different levels of the health system, not just in the US but also in developing countries. It provided us with a contrast and a real world example to work through difficult issues.”

Following the session, the small-group facilitators met with the course director to debrief. The feedback from the facilitators was overwhelmingly positive. They indicated that the small-group discussions were lively, that the case presented plausible challenges, and that it caused the learners to think critically about these issues. Timing for the parts of the case was generally adequate. Feedback from faculty who facilitated the small groups indicates that the facilitator notes were very useful.

## Discussion

This case study was developed as part of a larger Health Systems course at the Pennsylvania State University College of Medicine for first-year students in order explore the socioeconomic determinants of health through a global health lens. Initially, the presentation was designed to be given as a lecture, but the method was modified in order to engage all students more thoughtfully in the discussion.

Through this case, students are challenged with the startling fact that an adolescent boy's heart condition was entirely preventable with antibiotics; however, lacking access to the most basic of health care services in rural Haiti, he nearly died. Furthermore, the students are asked to imagine themselves in the future playing a part in this boy's care. Since the case study begins in a clinic in the US, all students are capable of contributing, not simply those with some prior global health experiences.

The strength of this approach is that students first engage with the material in their small groups, wrestling with some ethical concerns as well as learning from one another's knowledge and insight. The small group provides a setting in which all students can be encouraged by facilitators to participate. Students come to the group with prior knowledge and experience along a spectrum related to the topic. All are encouraged to share their thoughts with each other. Shifting toward a larger group setting for the second half then requires the students to continue to discuss amongst themselves in their small groups while sharing their opinions with other groups. In this way, the learning expands and deepens further, and insightful comments are not confined to one small group.

One weakness of this case study is the need to conclude the session with a discussion rooted more firmly in the domestic context. While the session ends with an open discussion based on the learning objectives, concluding with concrete examples from the domestic context would likely strengthen the relevance of the material for students’ future practice in the US. This will be a focus of future revisions.

Another weakness is the format of the large-group session, which relies on my personal experiences. For the purposes of peer review, the large-group session has been redesigned from its original form, which consisted of slides where I described my personal experiences in rural Haiti. Appendix C is now a more general set of slides that can guide any large-group facilitator through the material, but future facilitators are encouraged to add additional experiential context.

An important distinction between this global health case study and most case studies I have encountered is in the focus of the objectives. Most global health case studies develop educational objectives that explicitly highlight global health learning. This case study illustrates crosscutting medical education themes that align with LCME guidelines and situates them in both domestic and international settings, drawing lessons from both. The domestic elements of the case attempt to draw students who otherwise might dismiss global health as irrelevant to future practice into the discussion.

While I am aware of a variety of online free resources to teach global health topics, there is a need to develop medical education content that includes global health learning applicable to all students, not just those highly interested in global health. By anchoring the cases in real-world experiences, situated at least in part in a US context, educational opportunities such as this case study may provide all students with a greater sense of relevance and applicability to future practice. This approach, in turn, has potential to open up global health lessons for all students, not just those who pursue global health out of personal interest.

## Appendices

A. Small-Group Case Study - Facilitator.docx
B. Small-Group Case Study - Student.docx
C. Large-Group Slides.pptx
D. Large-Group Facilitator Guide.docx
E. Additional Case Details.pdf All appendices are peer reviewed as integral parts of the Original Publication.
