# Supplementary material for: Exploring Health Systems Within the Context of Social Determinants of Health: A Global Health Case Study
Source: MedEdPORTAL. 2016 Sep 23;12:10457. doi: 10.15766/mep_2374-8265.10457 (PMC6464409; doi:10.15766/mep_2374-8265.10457)
Supplement: Supplementary file 1 — A. Small-Group Case Study - Facilitator.docx B. Small-Group Case Study - Student.docx C. Large-Group Slides.pptx D. Large-Group Facilitator Guide.docx E. Additional Case Details.pdf [file mep-12-10457-s001.zip › E. Additional Case Details.pdf]

Community

People in the rural villages of Bon Samaritan rely heavily on one another. They will pool their resources together in times of need.

They are religious, with Catholicism being the dominant religion.

Most adults are either illiterate or have up to a 3rd grade education. There is no High School in the entire region.

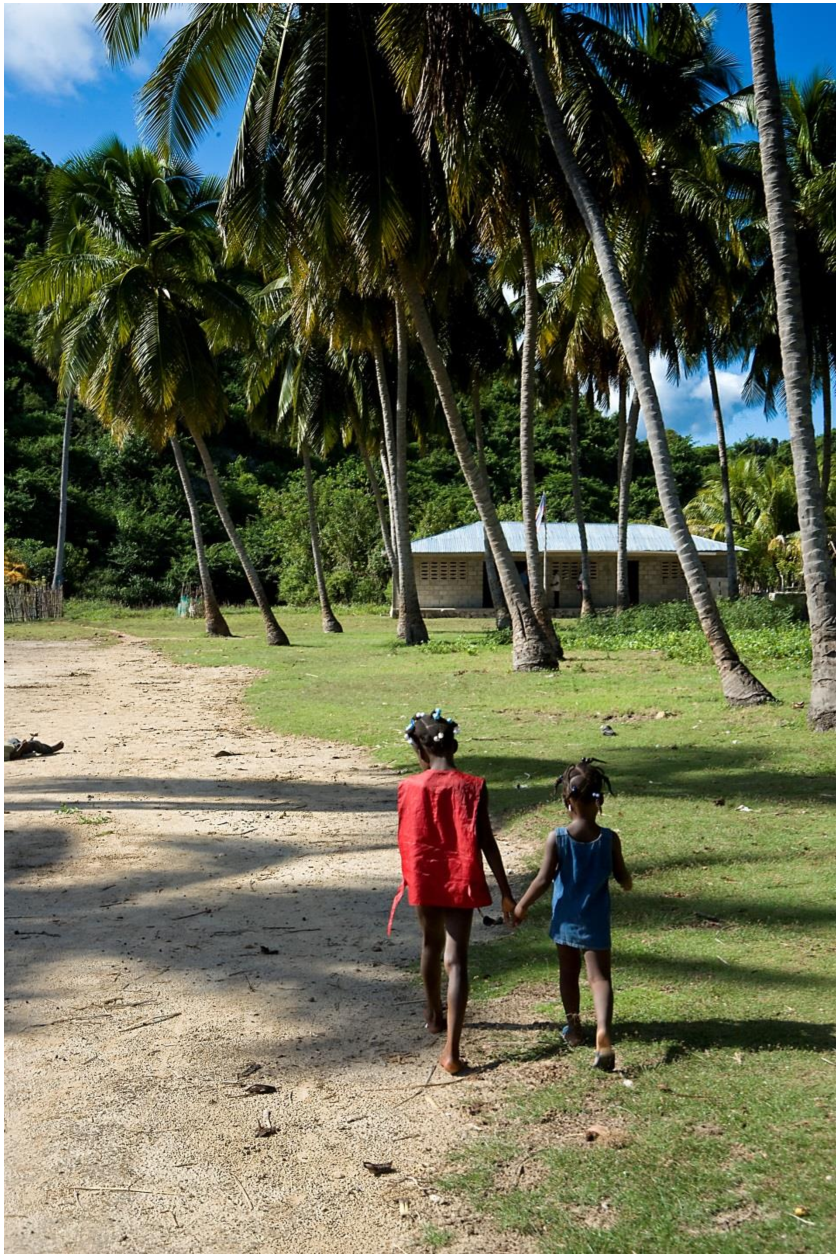

Image by Steve Miller, used with permission

Environment

Situations as seen in this photo taken in Bon Samaritan are breeding grounds for disease (there is a hog in the water).   The limited water they do have is contaminated (through open defecation by humans and animals), teeming with mosquitos that carry malaria and dengue fever.

Without access to vaccines tetanus is a very real and life-threatening problem.

Lack of trees (due to deforesting) results in topsoil being washed into the ocean with each heavy rain.

Major infectious disease include malaria, typhoid, intestinal parasites, dengue fever, and TB. Injuries like broken bones, water accidents (e.g. drowning), and burns are very difficult to manage and result in significant disability or early death.

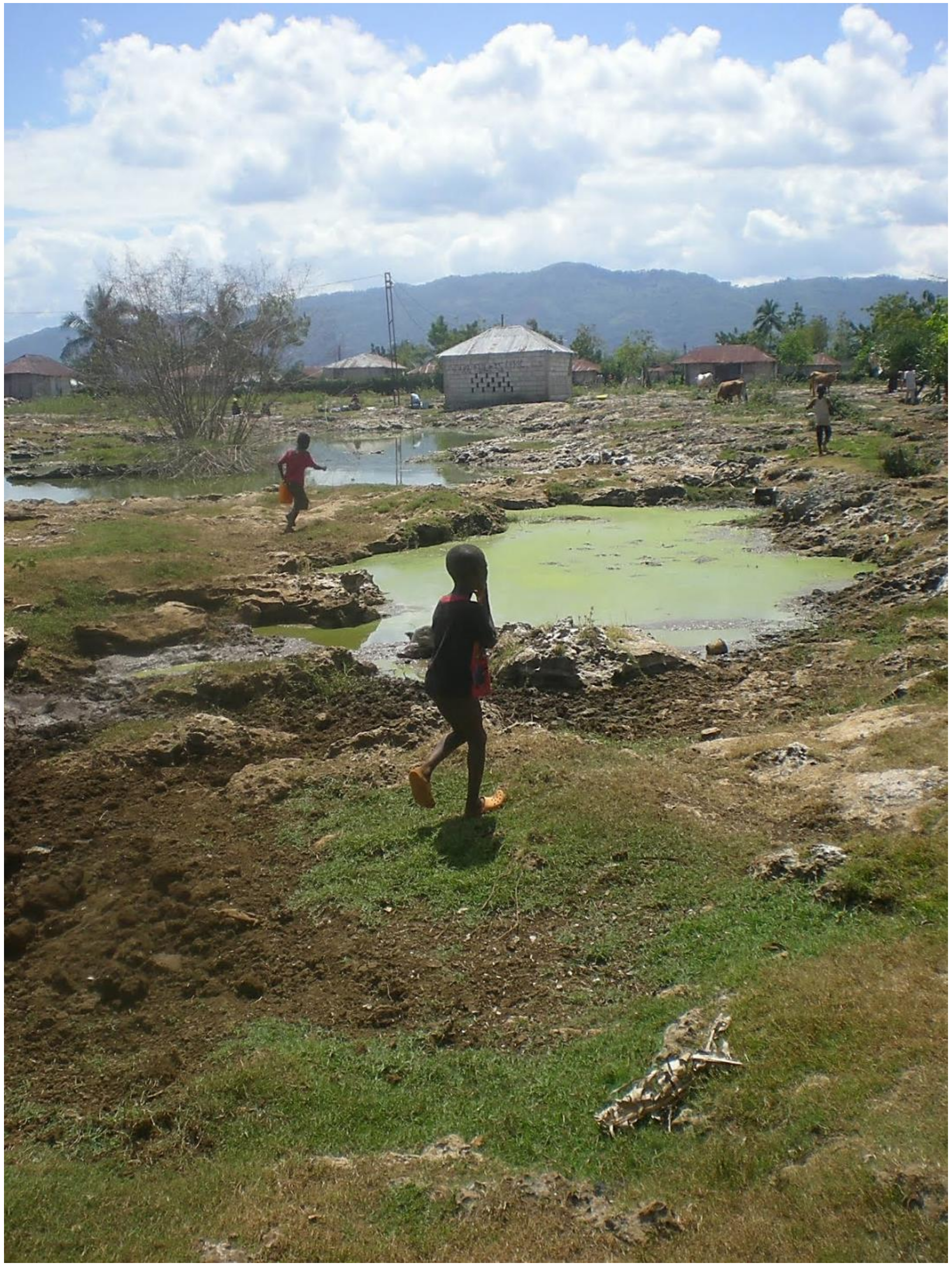

Image by Steve Miller, used with permission

Transportation

In Bon Samaritan, since most do not have vehicles, people walk to wherever they are going.   About 1/3 of the population live in villages reachable only by narrow footpaths. When radiators blow, tires go flat, or electrical problems arise ,vehicles are difficult to repair because of a lack of automotive expertise.

Kids typically walk to school about 1-2 hours one-way.

Transportation to the cities via overloaded buses or boats is available but crowded, and can be very unsafe.

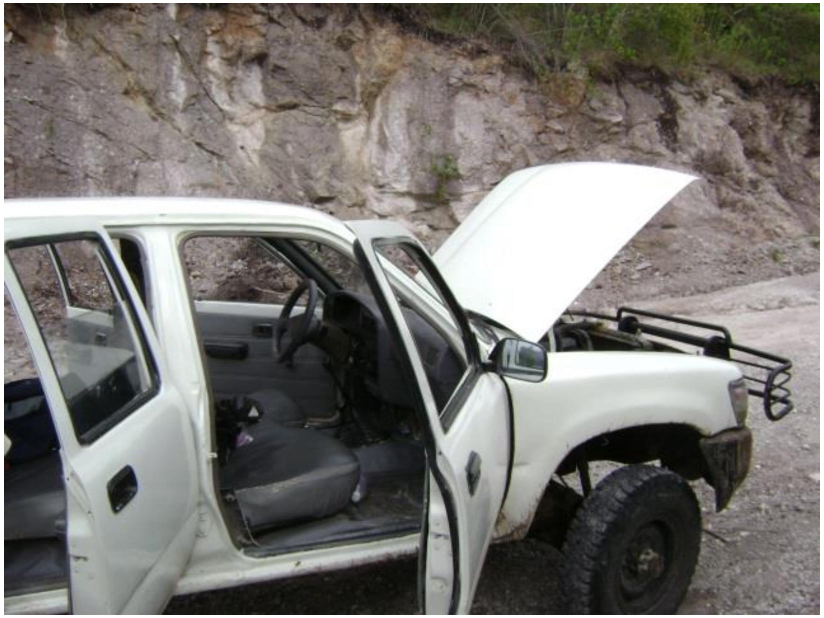

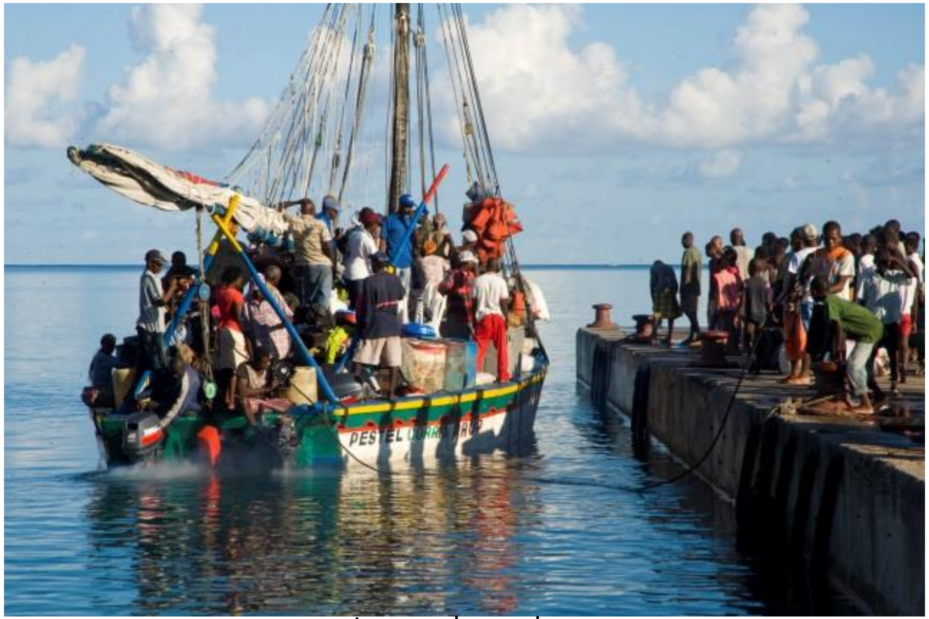

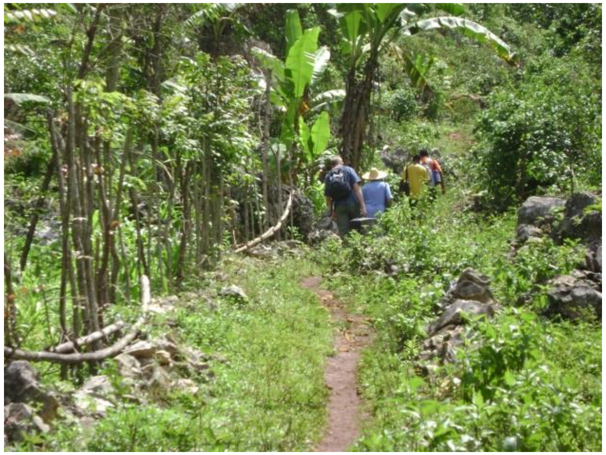

Images by author

Rural Housing

The average household in rural Bon Samaritan includes 6 people in a two room building. Generally the people are subsistence farmers, selling what they grow to purchase items (e.g. cooking needs, cell phone cards, education expenses, etc.).

Sanitation is by open defecation, as occur still in 15% of the world's population.

There is no electricity.

Cooking over an open fire is typically done in an out-building like this one, or inside a house. Severe burns occur when children fall into the fire. Smoke inhalation contributes to lung disease.

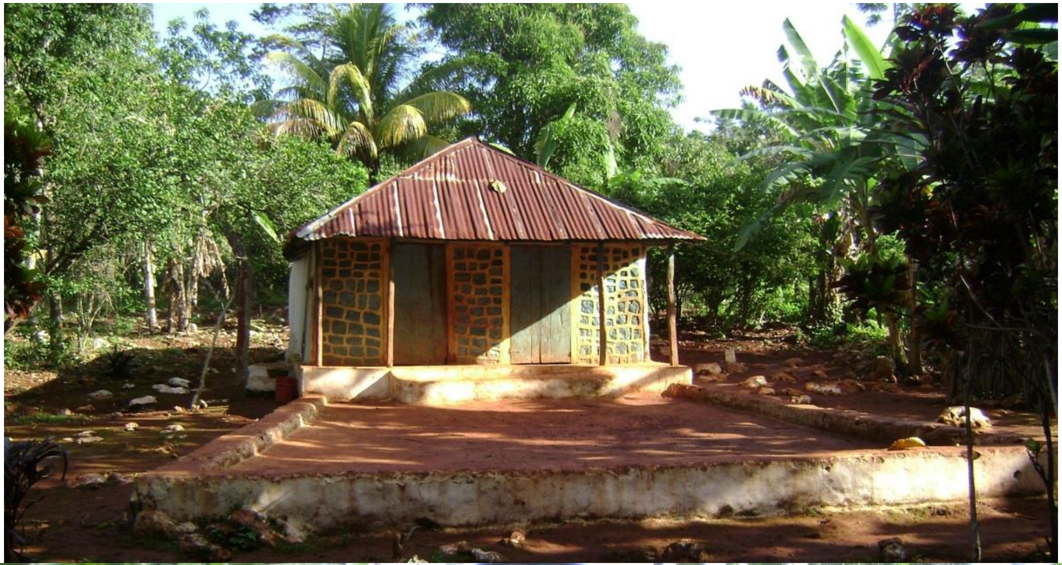

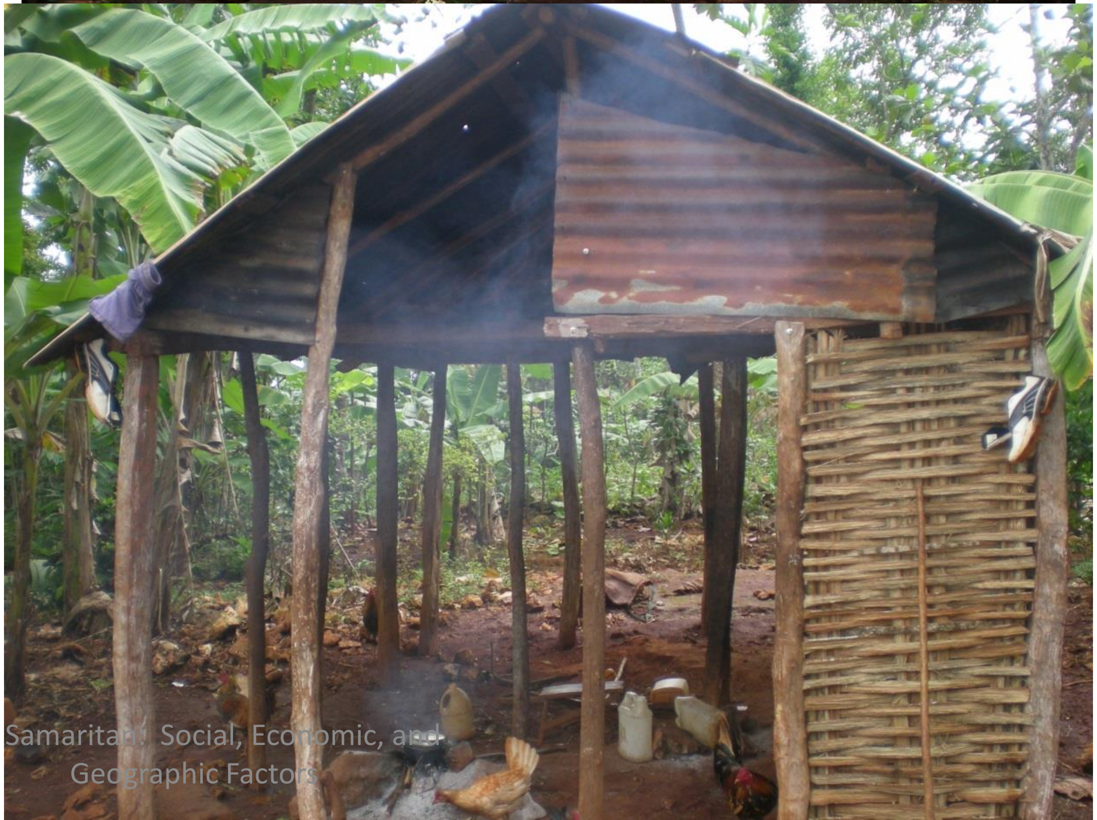

Images by author

Water Deprivation

In Bon Samarian very few groundwater sources like ponds exist. Most people will collect water in buckets or concrete cisterns when it rains, or they will haul water back in buckets.

Bon Samaritan is severely water deprived by World Health Organization standards (>15 minutes walk one way to obtain water). The #2 cause of child death (ages 1-5) in Bon Samaritan is diarrhea. Furthermore, the water itself is also very contaminated and is a major source of illness and death.

A cholera outbreak in Haiti also swept through Bon Samaritan, killing many for lack of clean, safe water and access to IV fluids (which, while available in the health center during the outbreak, were not accessible to most due to location of the clinic by the coast)

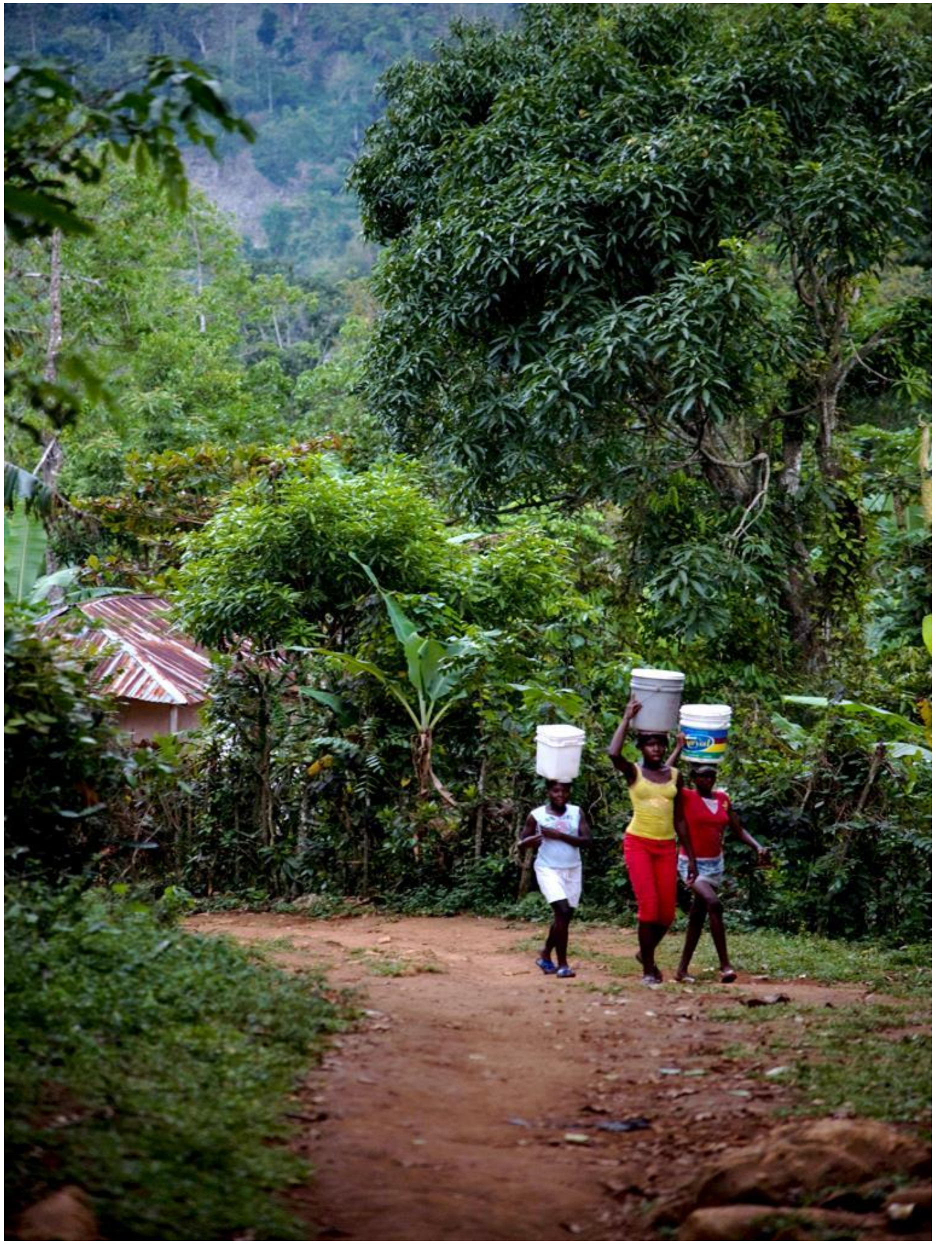

Image by Steve Miller, used with permission

Health Center in Bon Samaritan

Staffed by one Haitian physician and six nurses, the health center offers some basic primary care services (when medicines and supplies are available). However, regular shortages of supplies as well the center's location by the coast make health care generally inaccessible to the vast majority in this region.

The health center does have consistent electricity from solar power. However, the physician lacks human personnel to deliver vaccines and other Ministry of Health campaigns like high-dose Vitamin A to the region.

A health center in the mountains could make health care more accessible to thousands of people.

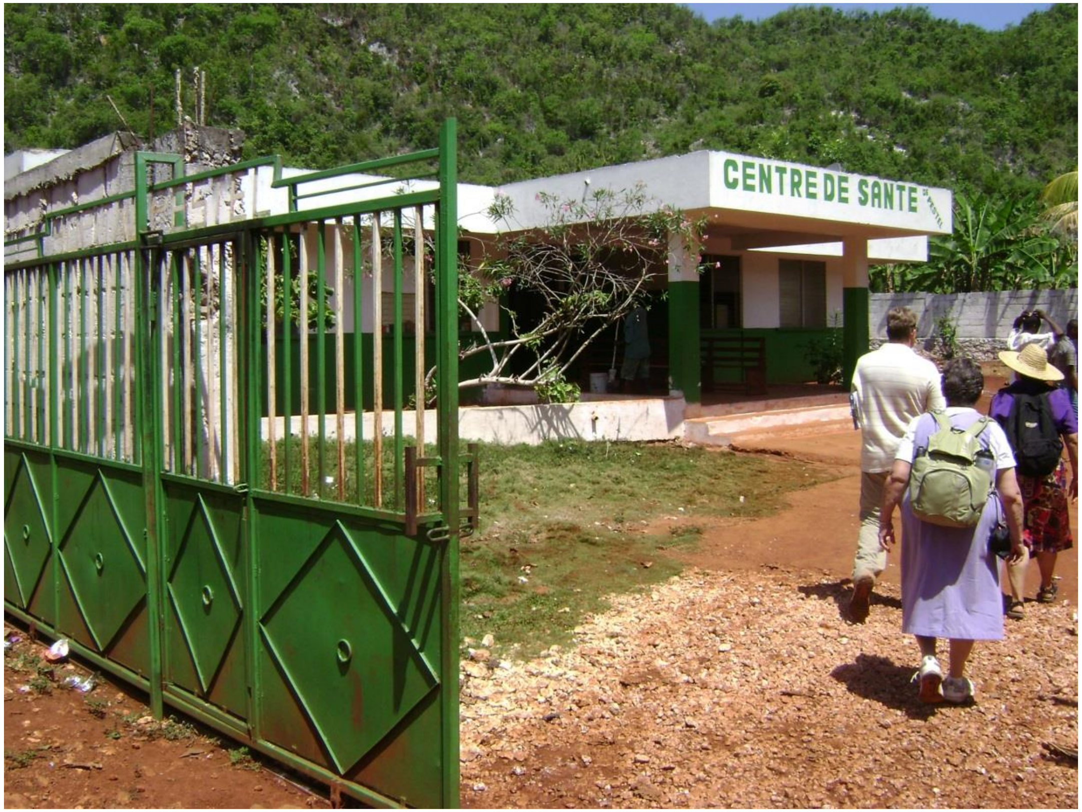

Image by author

Nutrition

This 7 month old child measures in the Orange Zone on the Mid-Upper Arm Circumference band. This is indicative of moderate malnutrition. His hair is thin and lightened.

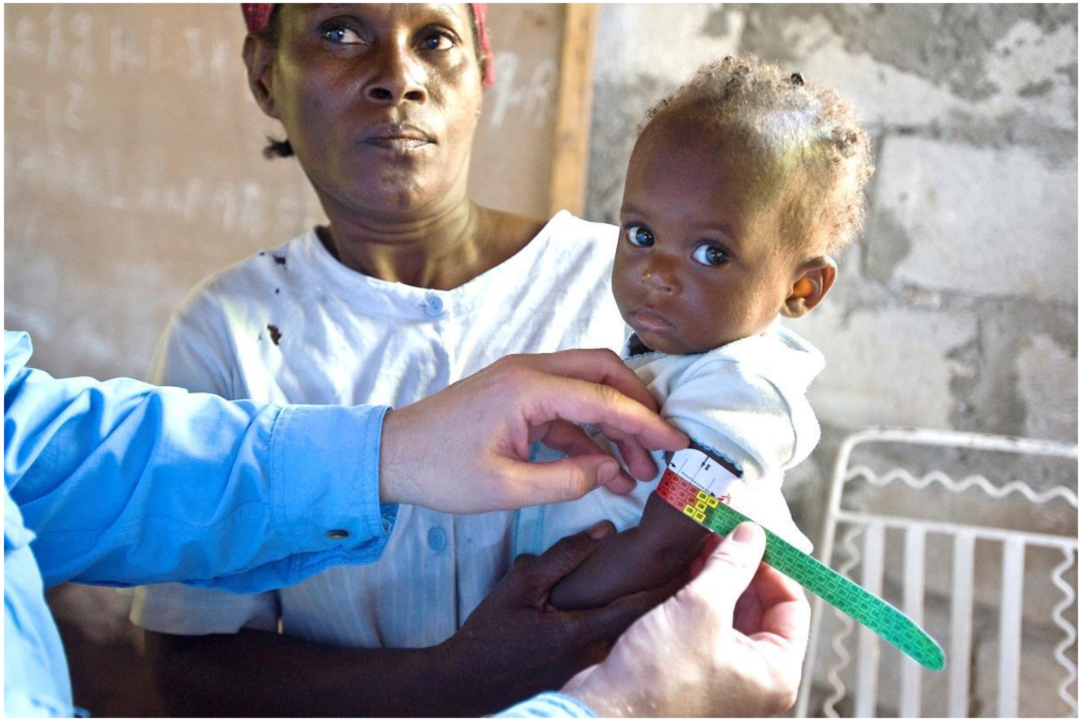

Malnutrition in this setting is indicative of deprivations at multiple levels (nutrition, sanitation, economic, health care, clean water). With the average daily income around $1US, families have little to spend on necessities.

~20% of children in Bon Samaritan are malnourished. 6% are severely malnourished.

High-dose Vitamin A twice annually to children in places like rural Haiti reduces all-cause child mortality by 20%.

Image by Steve Miller, used with permission

Bon Samaritan Health Care System

Governance is provided loosely by the Ministry of Health. They rarely make an appearance or check up on the health center.

Information is gathered by paper and stored at the health center. Reports are filed periodically with the Ministry of Health.

Financing is partly through the Ministry of Health (salaries) but any supplies, medicines, and fees associated with consults are all born by the patient. An obstetrical delivery at the health center costs $4US, for example.

Very few services are delivered outside the health center. The health center provides acute visits, hospital stays, obstetrical deliveries, HIV and TB case management. Very few use the available services for lack of money or distance .

Human resources are limited, and the potential to recruit locals is also limited because of the lack of educational opportunity throughout the region.

Technologies include basic microscopy, testing for HIV, and urinalysis. IV fluids and antibiotics are available at the health center.

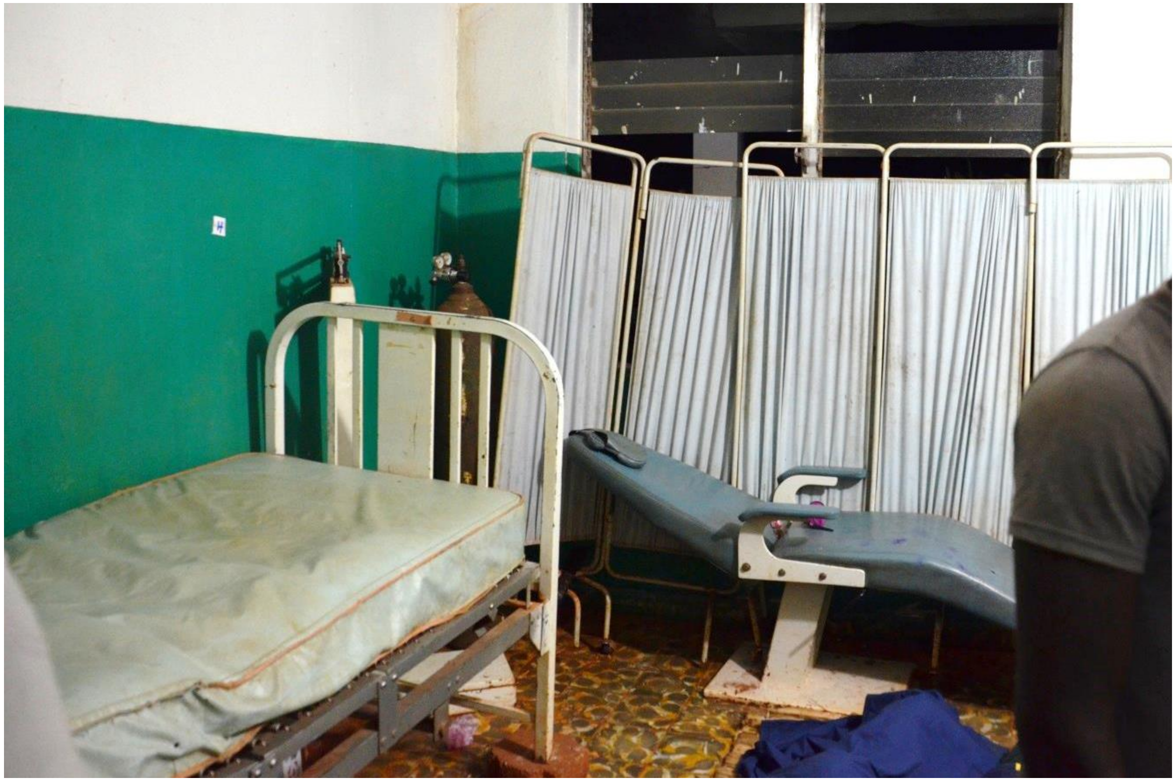

Image by James Bishop, used with permission
