# Supplementary material for: Exploring Health Systems Within the Context of Social Determinants of Health: A Global Health Case Study
Source: MedEdPORTAL. 2016 Sep 23;12:10457. doi: 10.15766/mep_2374-8265.10457 (PMC6464409; doi:10.15766/mep_2374-8265.10457)
Supplement: Supplementary file 1 — A. Small-Group Case Study - Facilitator.docx B. Small-Group Case Study - Student.docx C. Large-Group Slides.pptx D. Large-Group Facilitator Guide.docx E. Additional Case Details.pdf [file mep-12-10457-s001.zip › B. Small-Group Case Study - Student.docx]

**Global Health Case Study Small Group Session**

**STUDENT Version**

**KEY MESSAGE**: *An individual’s health and ability to seek health care services are affected by a complex interplay of social, economic and environmental factors within the community, often dramatically illustrated in underserved settings. Health systems are a community resource for health, and the availability of even basic health systems can have significant impact on individual and population health.*

**Objectives**

**By the end of the session, students will be able to:**

1. Demonstrate an awareness of the larger social and economic context in which health systems exist.
2. Recognize the importance of viewing an individual’s health within the context of community resources, including the availability of health systems.
3. Discuss how a patient’s capacity to pursue health care services is affected by the larger social and economic context.

**Small Group Activity (PBL): 50 MINUTES**

1. Timeline for Small Group:
   1. Hand out part 1 to the group (10 minutes)
   2. Hand out part 2 to the group (10 minutes)
   3. Hand out part 3 to the group (30 minutes)

**Large Group Activity: ONE HOUR**

**Health Systems in a Global Context**

**Student Version**

|  | Under-5 Mortality rate^[[1]](#footnote-1)^,^[[2]](#footnote-2)^ | Maternal mortality ratio^[[3]](#footnote-3)^ | Life expectancy at birth^[[4]](#footnote-4)^ |
| --- | --- | --- | --- |
| Haiti | 69 | 359^[[5]](#footnote-5)^ | 63 |
| World | 48 | 216^[[6]](#footnote-6)^ | 70 |
| United States | 7 | 14^[[7]](#footnote-7)^ | 79 |

*Time allotment for this page: 10 minutes*

**HAITI FAST FACTS**

**Part 1 of 3**

**12 years in the future when you are in your practice….**

You are approached by some good friends, Doug and Sandy, who recently began serving as host parents for a twelve year old Haitian boy named Jean Baptiste who was brought to your hospital for free heart surgery. They ask if you will help them with some pre-op and post-op medical issues, should any arise. You agree to provide this as a free service through your primary care office.

You review a clinic note from a pediatric cardiologist:

“Thank you very much for the opportunity to evaluate Jean Baptiste in consultation. As you may recall, he is a 12-year-old boy who has been here in the United States for less than 12 hours (i.e. at the time of this evaluation). He is seen in the company of his host family representative (i.e. Sandy). Jean Baptiste speaks primarily Creole with some element of French. Consequently, there is no apparent information regarding any significant medical problems outside of his present cardiovascular concerns. His remaining review of systems and social history are non-contributory. He is receiving no medications.

Examination:

HR 81 and regular BP 100 systolic/68 diastolic SPO_2_ room air 99%

In general, Jean Baptiste was a somewhat tall for age and thin black male in no distress. He was acyanotic. There was no jugular venous distention. His lungs were clear to auscultation. On cardiac examination, S1 and S2 were present. There was a grade 2 to 3/6 blowing, long systolic murmur heard best at the apex. This murmur radiated to the anterior axillary line on the left, the left axilla, the left sternal border, and faintly into the left hemithorax. A prominent lift was present at the left lower sternal border. The PMI was displaced well into the lateral aspects of the left hemithorax. Peripheral pulses were of normal character and equal intensity. There were no palmar pulses. The liver edge was palpable at the right costal margin in the mid clavicular line. There was no abdominal bruit. The capillary refill was brisk. Maneuvers to elicit findings of mitral valve prolapse were nonproductive. The remainder of the cardiovascular examination was within normal limits.

ECG: 1) Normal sinus rhythm; 2) left atrial enlargement; 3) left ventricular hypertrophy

**QUESTION**: Discuss pertinent findings and develop a differential diagnosis. Based on the above information, what is the most likely diagnosis? How does Jean Baptiste’s country-of-origin affect your differential diagnosis?

**STOP.**

Part 2 of 3

*Time allotment for this page: 10 minutes*

Diagnosis: The patient has severe rheumatic mitral valve insufficiency. This could have been prevented with penicillin to treat his initial strep throat infection. Through a translator Jean Baptiste tells you that he could not see a doctor when he had a sore throat.

The underlying cause was untreated strep pharyngitis. This could largely have been prevented by oral antibiotics (penicillin). This patient did not have access to antibiotics because of the lack of basic medical care in rural Haiti.

Globally rheumatic heart disease is the leading cause of pediatric heart failure and cardiovascular death, and 80-85% of children worldwide live in areas where it is endemic. The disease, however, has essentially been eliminated in developed countries. It is now a disease of poverty.^[[8]](#footnote-8)^ “Acute rheumatic fever and rheumatic heart disease can be regarded as physical manifestations of poverty and social inequality.”^[[9]](#footnote-9)^

**QUESTION**: Jean Baptiste’s host parents want to understand how this boy could be so close to death for lack of access to a basic antibiotic like penicillin. What are THREE critical economic, geographic, or other health care access factors that affect the availability of health care delivery in under-resourced countries? Be specific in your answers.

**QUESTION**: Describe what is necessary at each step if you were to develop a strep throat infection. Then consider the World Health Organization’s “Framework for Health System Components” (below). What are the health system components that need to be in place in order for the relatively simple process to occur of ensuring that a child with a strep throat infection receives proper antibiotics?^[[10]](#footnote-10)^ ^[[11]](#footnote-11)^

Framework for Health System Components:

|  | Description |
| --- | --- |
| GOVERNANCE | WHO is responsible for oversight, supervision? |
| FINANCES | HOW is the work going to be funded? |
| PERSONNEL | WHO is doing the work? |
| SUPPLIES | WHAT supplies are necessary to provide the services? |
| INFORMATION | WHAT information needs to be collected, how is it collected, and who collects, reports, and analyzes it. |
| SERVICES | WHAT health services will be provided and WHY? |

**STOP.**

Part 3 of 3

Time allotment for these pages: 30 minutes

After recovering from a bio-prosthetic valve replacement, Jean Baptiste returns to rural Haiti.

Doug and Sandy are grateful for your assistance. They ask you to travel with them on a one-week trip to a remote region in Haiti called Bon Samaritan where Jean Baptiste lives with his parents. You decide to go with them on the trip.

Doug and Sandy have already received $100,000 in donations to be used to improve health of people living in the region of Bon Samaritan. In the large group session you will explore three potential health initiatives that might be started for this region with the $100,000 donation. By the end of the large group session your group will vote on which of the three health initiatives, if any, should be developed with the $100,000. The potential health initiatives for the region of Bon Samaritan are:

1. **New Clinic Building**
2. **Community Health Workers (CHWs) Program**
3. **Well-drilling Project**

Before your trip you read up a bit on Haiti. People living in rural Haiti are disadvantaged on multiple levels—typically there is no running water, electricity, toilets, no paved roads, emergency services, or basic health care such as safe childbirth or treatment for malaria.

**Brief description of the existing health system in Bon Samaritan, Haiti:**

- The vast majority of people in Bon Samaritan have no access to health care services. Very few people can access the one existing health center because of its location along the coast. For those who can access the health center, not much can be said about quality of care they receive because no one is measuring or evaluating this. Financing of health services is very challenging because the population is extremely poor and the government salary support for the six nurses and one Haitian physician is unreliable due to governmental mismanagement. This, in turn, affects the health center’s ability to purchase supplies and medications. Public health efforts are absent in this area.

**Map of the Region of Bon Samaritan.** Villages are shown as small dots. Jean Baptiste lives in one of the villages denoted as a green dot, about 2-3 hours walk one way to the dilapidated Health Center.


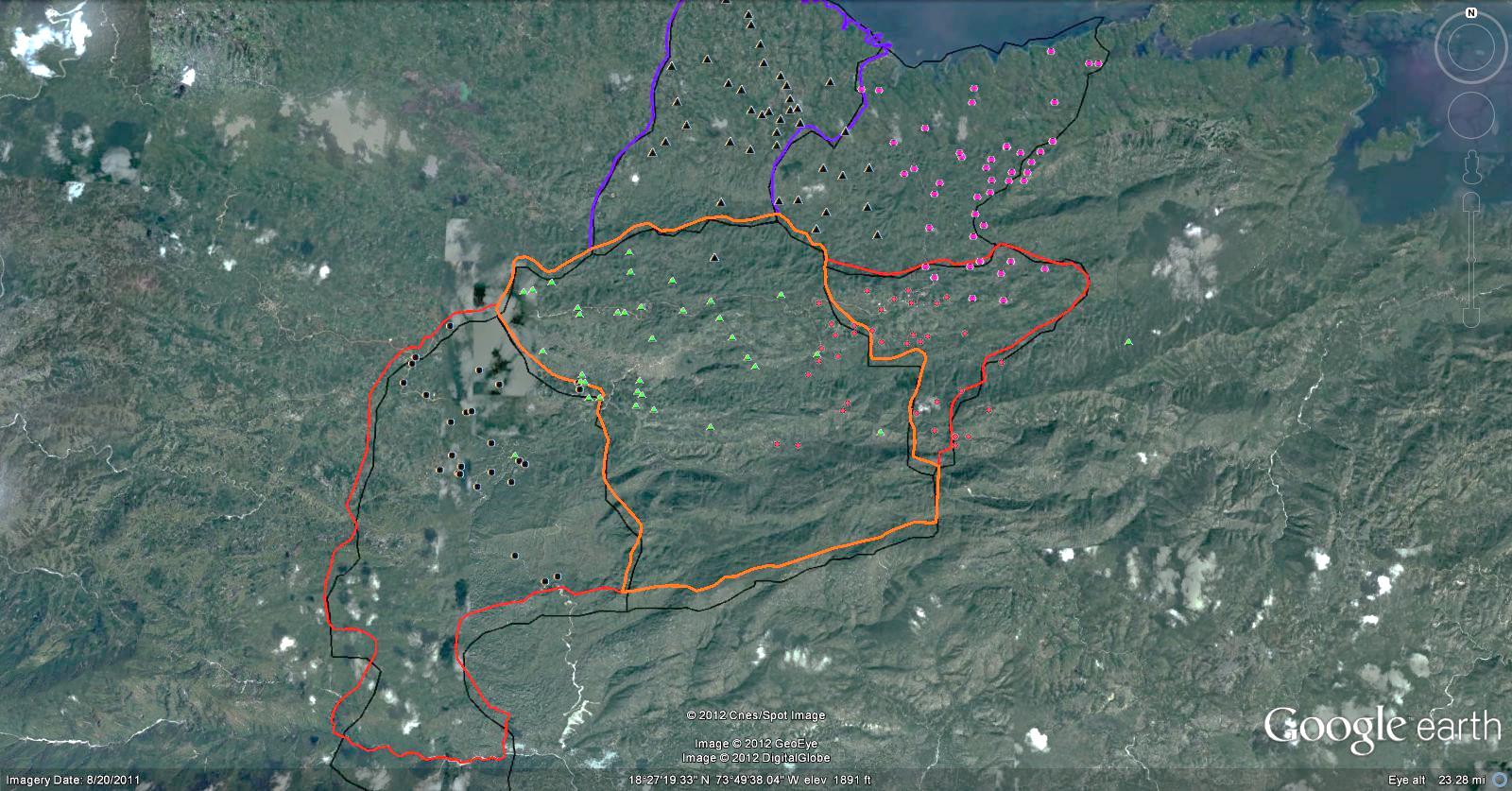

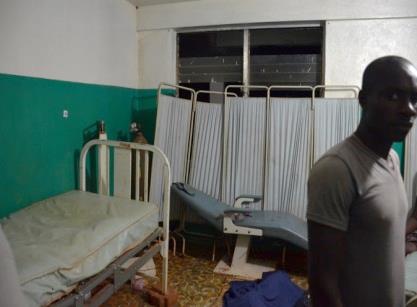


Health Center

Map image used by permission from Google Earth^[[12]](#footnote-12)^, “©2012 Cnes/Spot Image”, “Image©2012 GeoEye”, “Image©2012DigitalGlobe”.

Jean’s village

The Regional Health Director for Bon Samaritan has presented your group with three health initiatives that he believes would be beneficial to the Region of Bon Samaritan. At the end of the Large Group session your group will vote to use the $100,000 for one the following:

1. **New Clinic Building.** Build a new (unfurnished) clinic up in the mountains near Jean’s village to provide an access point to those without any health care access.
2. **Community Health Workers (CHWs)**. Start vaccinations and high-dose Vitamin A with deworming campaigns to reach about 10,000 children throughout the whole region for the next five years. CHWs can also provide health education in the villages. While in some contexts trained CHWs can treat some diseases, they will not be able to provide treatment in Bon Samaritan due to lack of funds for ongoing training.
3. **Well-drilling Project**. Undertake a well-drilling effort to establish 10 groundwater wells throughout the region. Bon Samaritan is considered severely water deprived. Diarrhea is the #2 cause of child death (ages 1-5 years).
4. **None of the above**. Or “Other”.

This is based on a true story. In light of what you know of Bon Samaritan, consider what more information about each of the above initiatives you want before making a decision. Write down these questions and bring them to the large group setting. Then **come as a group with a decision** about which of the choices above your group will vote for in the large group setting.

Your group will join the large group in the lecture hall. **END OF SMALL GROUP SESSION.**

1. Probability per 1,000 that a newborn baby will die before reaching age 5 [↑](#footnote-ref-1)
2. <http://data.worldbank.org/indicator/SH.DYN.MORT> [↑](#footnote-ref-2)
3. Number of women per 100,000 live births who die from any cause related to or aggravated by pregnancy or is management [↑](#footnote-ref-3)
4. <https://en.wikipedia.org/wiki/List_of_countries_by_life_expectancy> [↑](#footnote-ref-4)
5. <http://www.who.int/gho/maternal_health/countries/hti.pdf?ua=1> [↑](#footnote-ref-5)
6. <http://www.unfpa.org/publications/trends-maternal-mortality-1990-2015> [↑](#footnote-ref-6)
7. <http://www.who.int/gho/maternal_health/countries/usa.pdf?ua=1> [↑](#footnote-ref-7)
8. *Marijon E, et al. Rheumatic Heart Disease. The Lancet, 2012;379(9819):953-964.* [↑](#footnote-ref-8)
9. *Rothenbuhler M , et al. Active Surveillance for rheumatic heart disease in endemic regions. Lancet Glob Health, 2014;2(12):e717-26.* [↑](#footnote-ref-9)
10. <http://www.wpro.who.int/health_services/health_systems_framework/en/> [↑](#footnote-ref-10)
11. <http://www.who.int/alliance-hpsr/about/hpsr/en/> [↑](#footnote-ref-11)
12. <https://www.google.com/permissions/geoguidelines.html> [↑](#footnote-ref-12)
