# Supplementary material for: Exploring Health Systems Within the Context of Social Determinants of Health: A Global Health Case Study
Source: MedEdPORTAL. 2016 Sep 23;12:10457. doi: 10.15766/mep_2374-8265.10457 (PMC6464409; doi:10.15766/mep_2374-8265.10457)
Supplement: Supplementary file 1 — A. Small-Group Case Study - Facilitator.docx B. Small-Group Case Study - Student.docx C. Large-Group Slides.pptx D. Large-Group Facilitator Guide.docx E. Additional Case Details.pdf [file mep-12-10457-s001.zip › C. Large-Group Slides.pptx]

## Slide 1
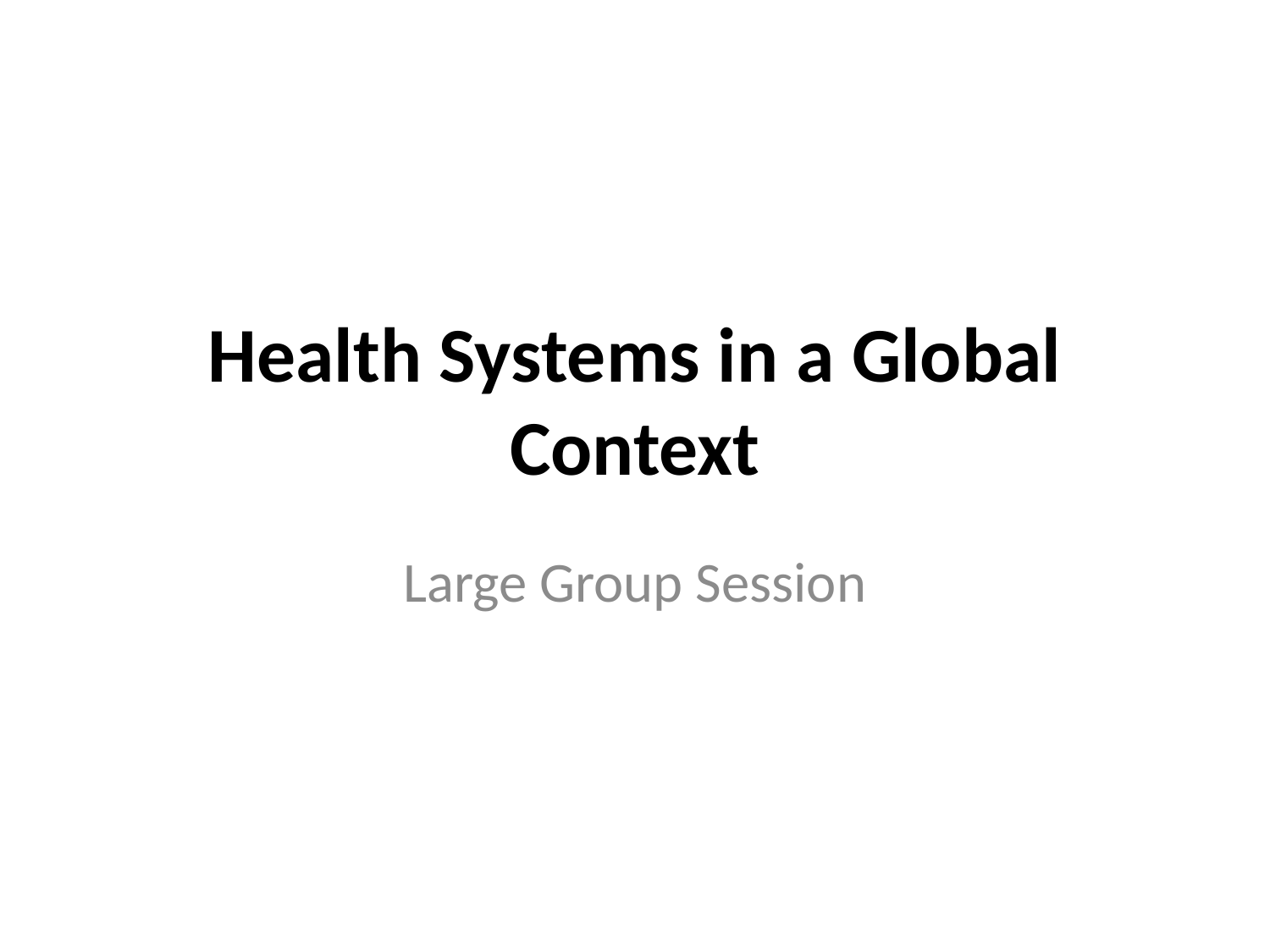

# Health Systems in a Global Context
Large Group Session

## Slide 2
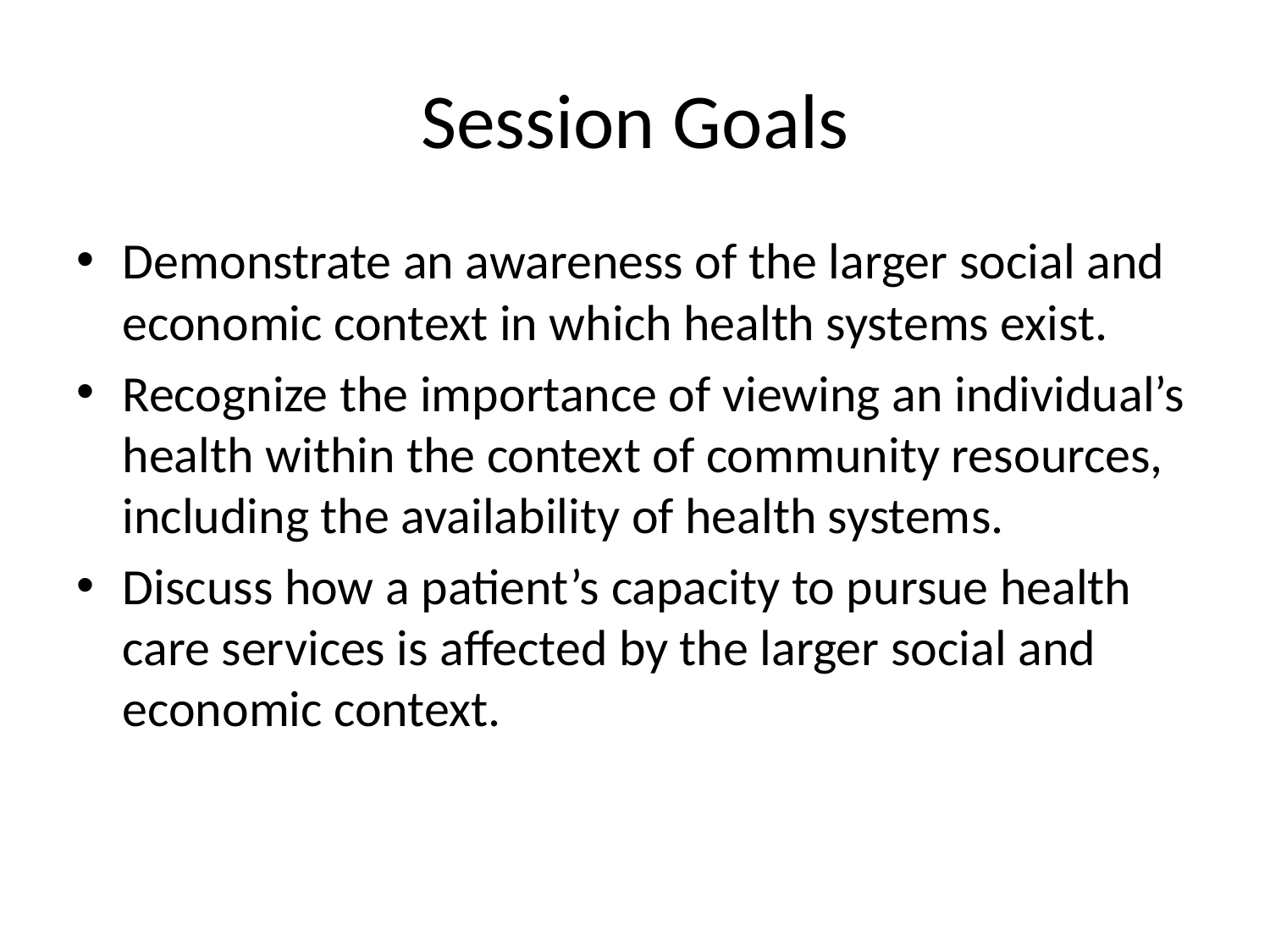

# Session Goals
Demonstrate an awareness of the larger social and economic context in which health systems exist.
Recognize the importance of viewing an individual’s health within the context of community resources, including the availability of health systems.
Discuss how a patient’s capacity to pursue health care services is affected by the larger social and economic context.

## Slide 3
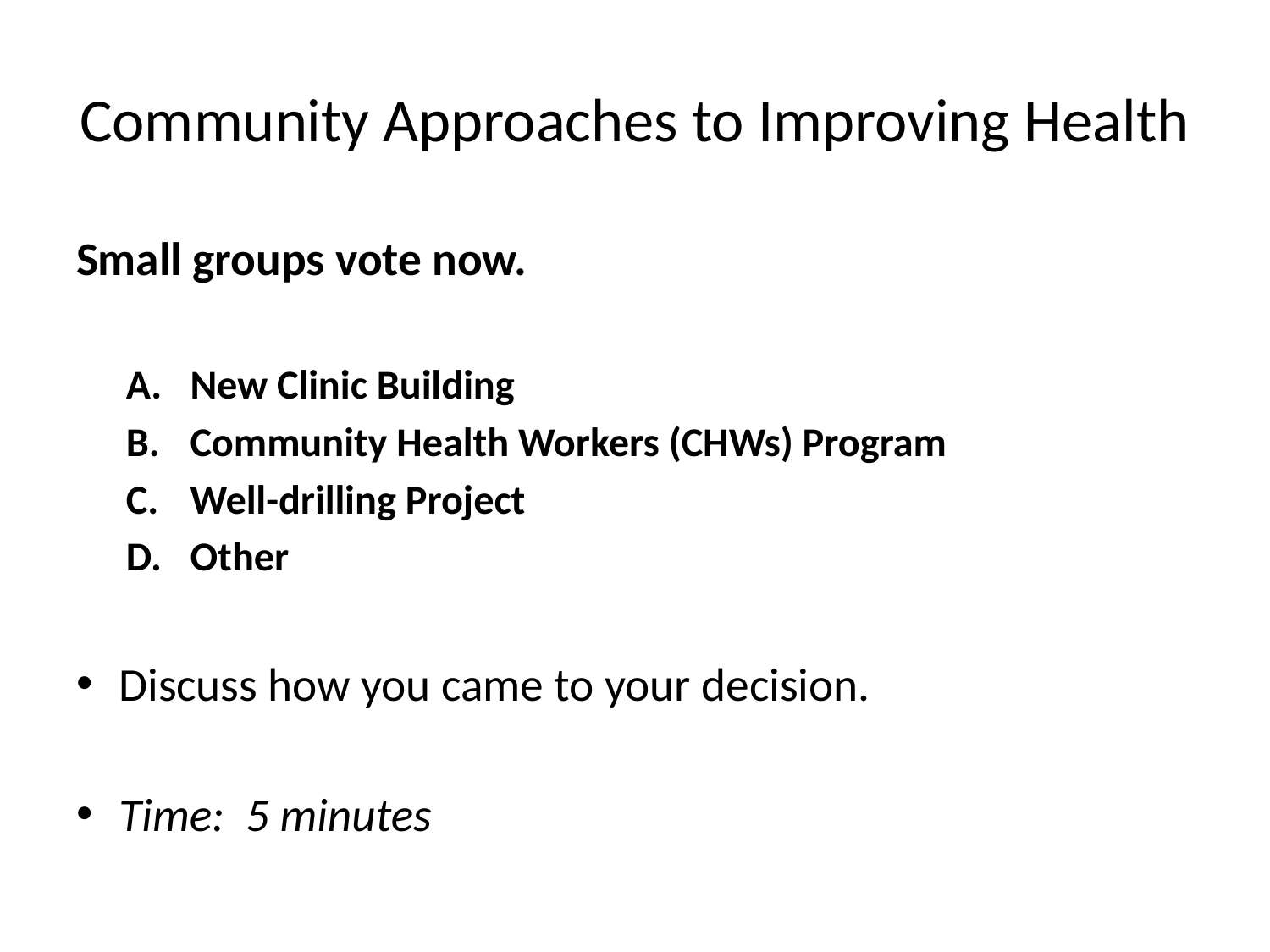

# Community Approaches to Improving Health
Small groups vote now.
New Clinic Building
Community Health Workers (CHWs) Program
Well-drilling Project
Other
Discuss how you came to your decision.
Time: 5 minutes

## Slide 4
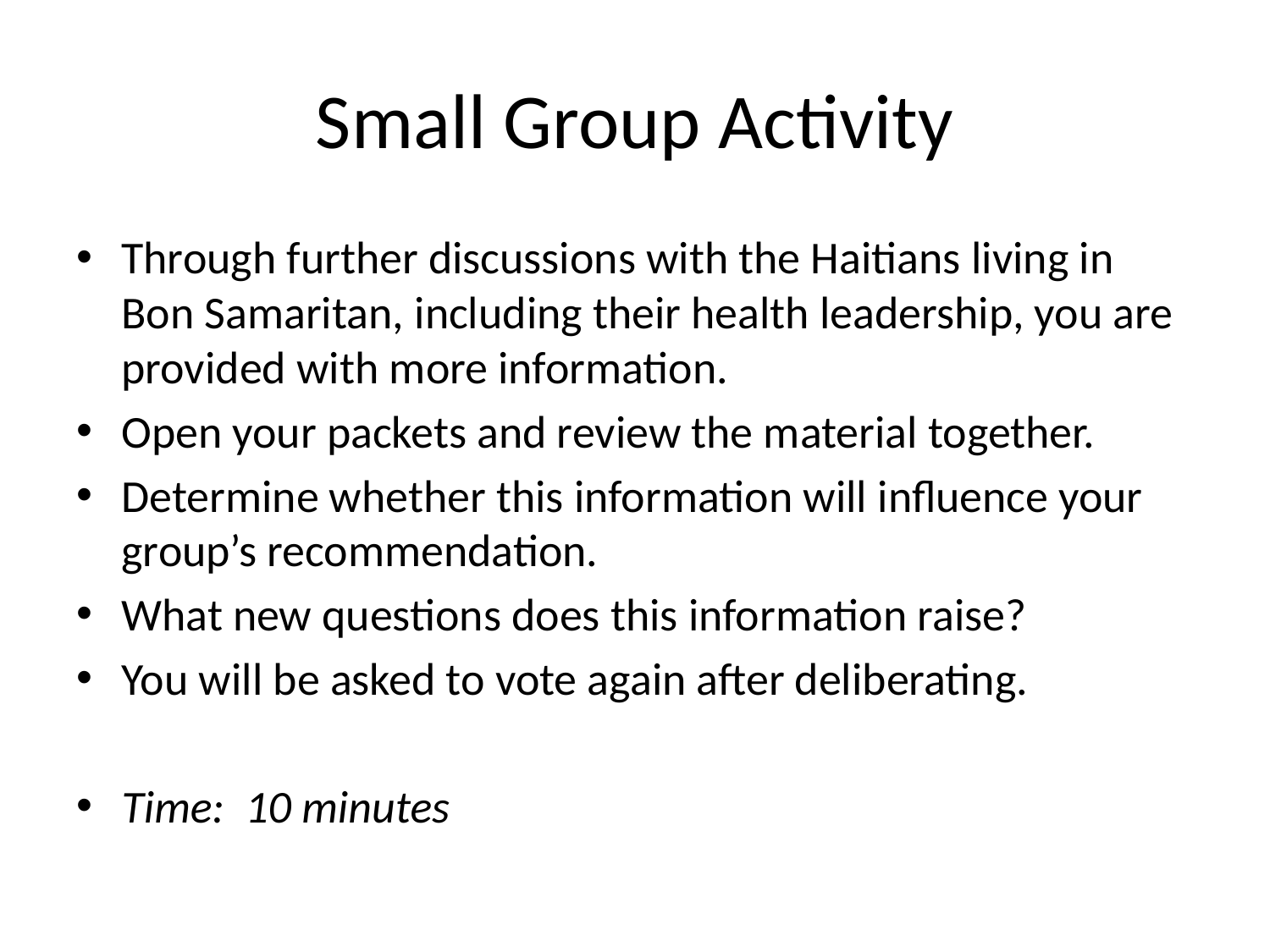

# Small Group Activity
Through further discussions with the Haitians living in Bon Samaritan, including their health leadership, you are provided with more information.
Open your packets and review the material together.
Determine whether this information will influence your group’s recommendation.
What new questions does this information raise?
You will be asked to vote again after deliberating.
Time: 10 minutes

## Slide 5
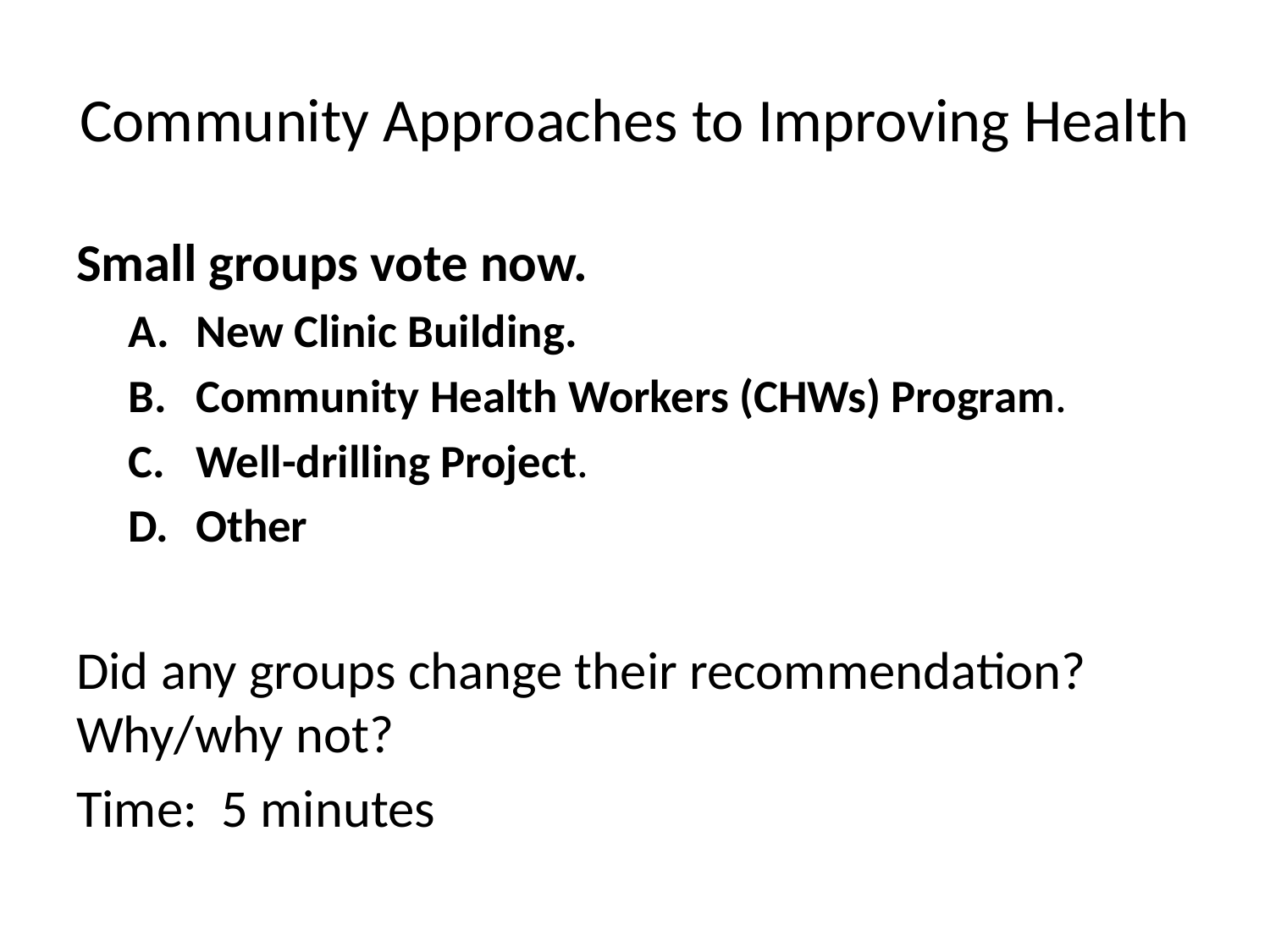

# Community Approaches to Improving Health
Small groups vote now.
New Clinic Building.
Community Health Workers (CHWs) Program.
Well-drilling Project.
Other
Did any groups change their recommendation? Why/why not?
Time: 5 minutes

## Slide 6
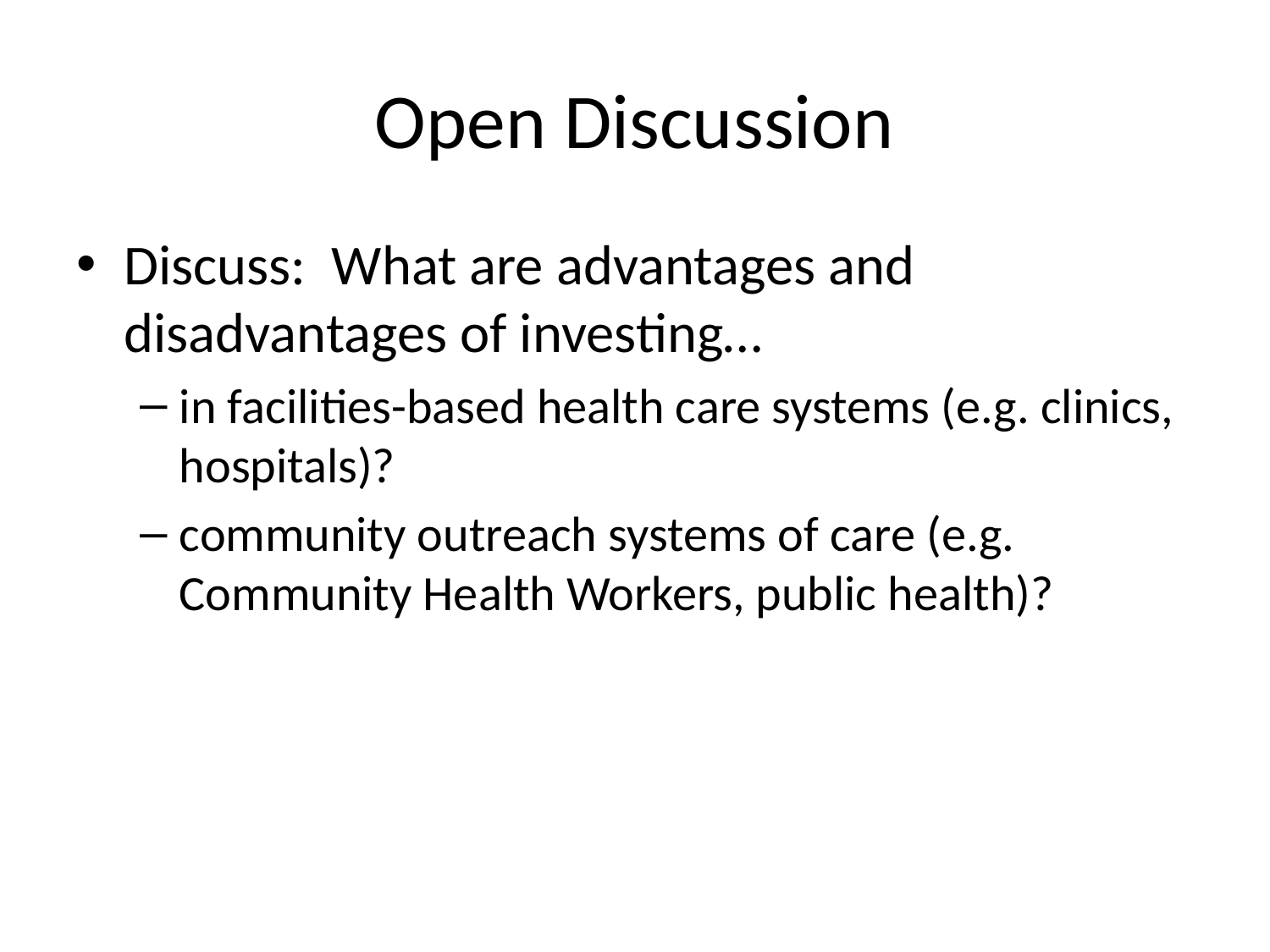

# Open Discussion
Discuss: What are advantages and disadvantages of investing…
in facilities-based health care systems (e.g. clinics, hospitals)?
community outreach systems of care (e.g. Community Health Workers, public health)?

## Slide 7
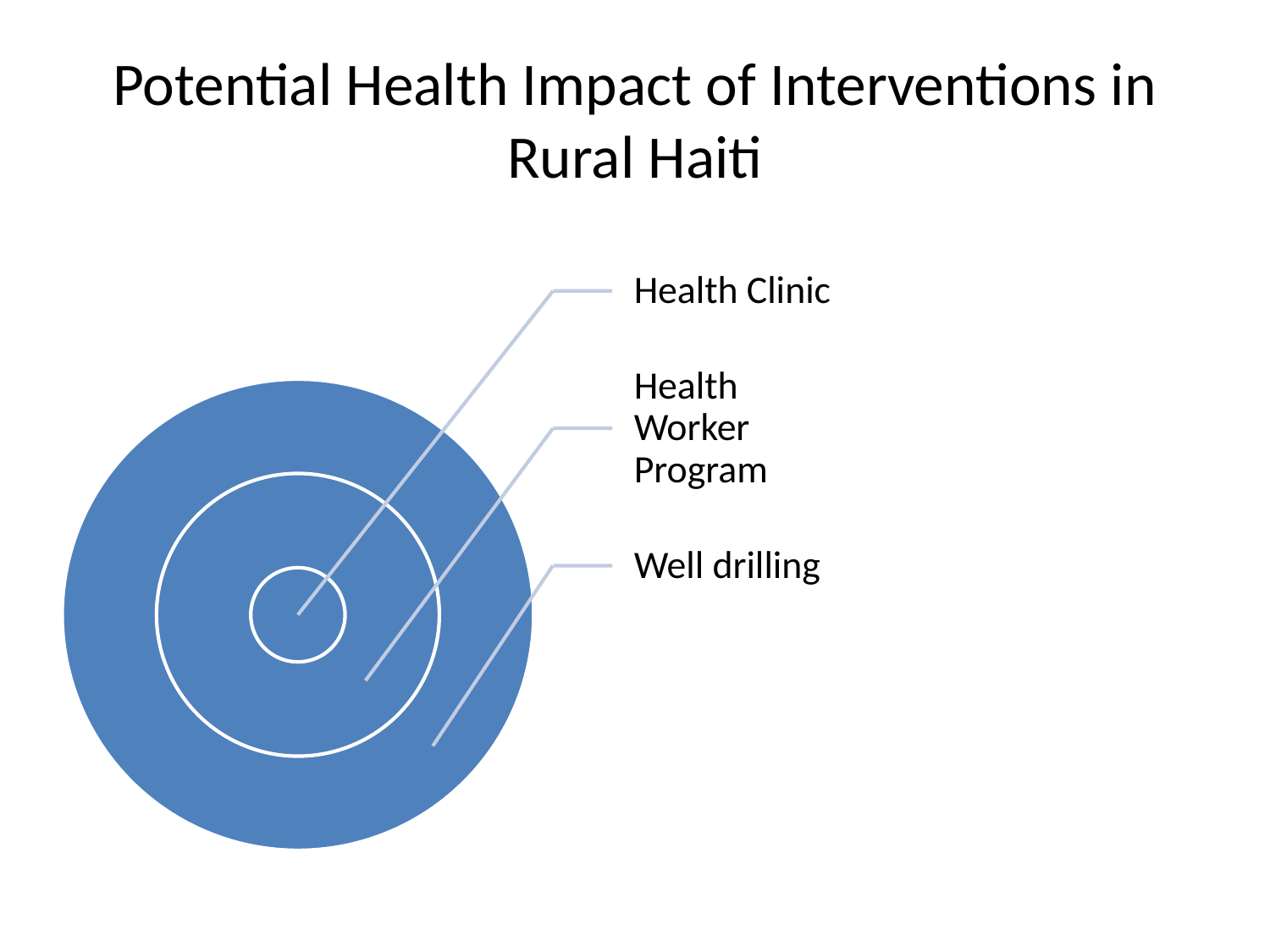

# Potential Health Impact of Interventions in Rural Haiti

## Slide 8
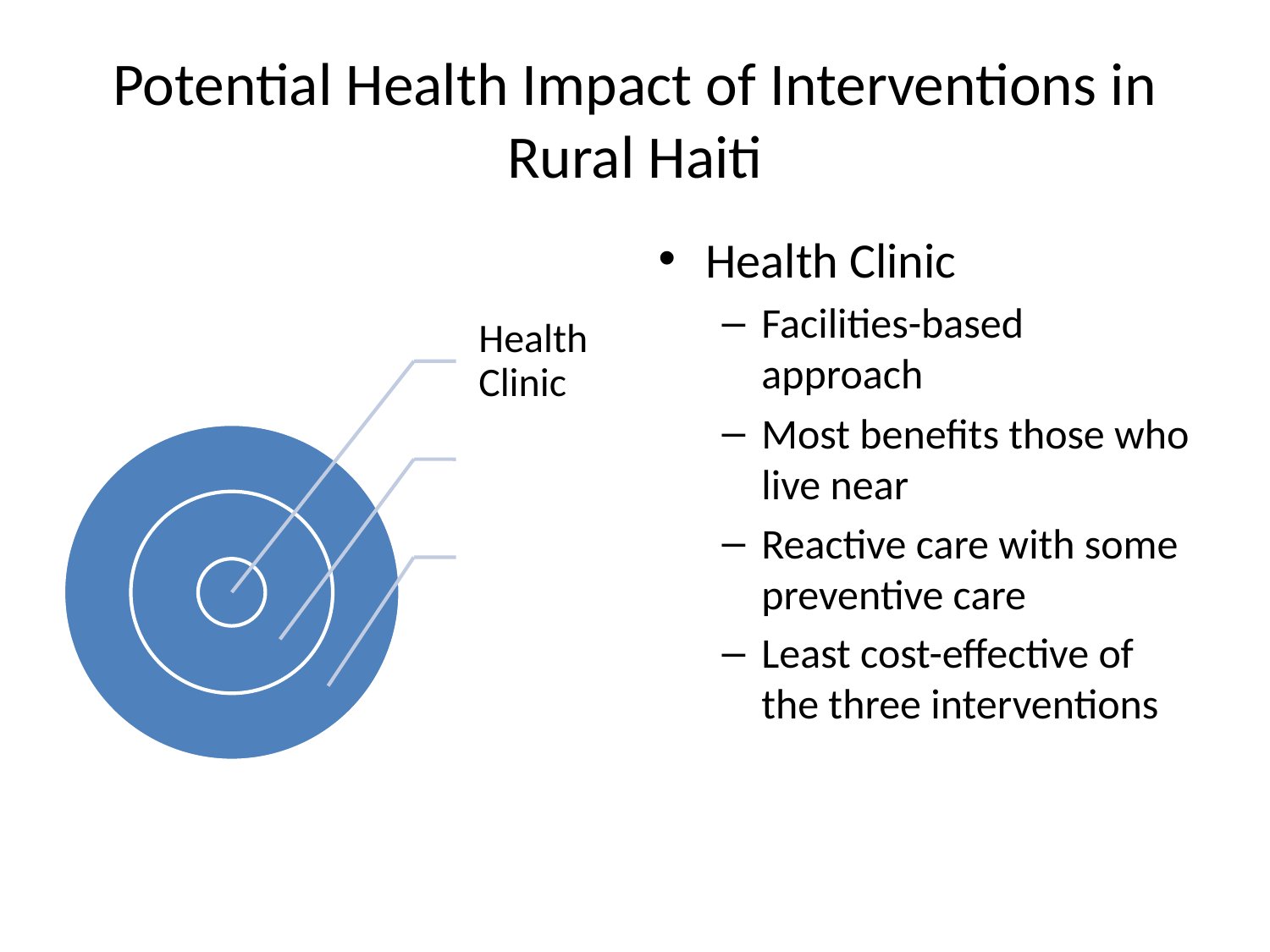

# Potential Health Impact of Interventions in Rural Haiti
Health Clinic
Facilities-based approach
Most benefits those who live near
Reactive care with some preventive care
Least cost-effective of the three interventions

## Slide 9
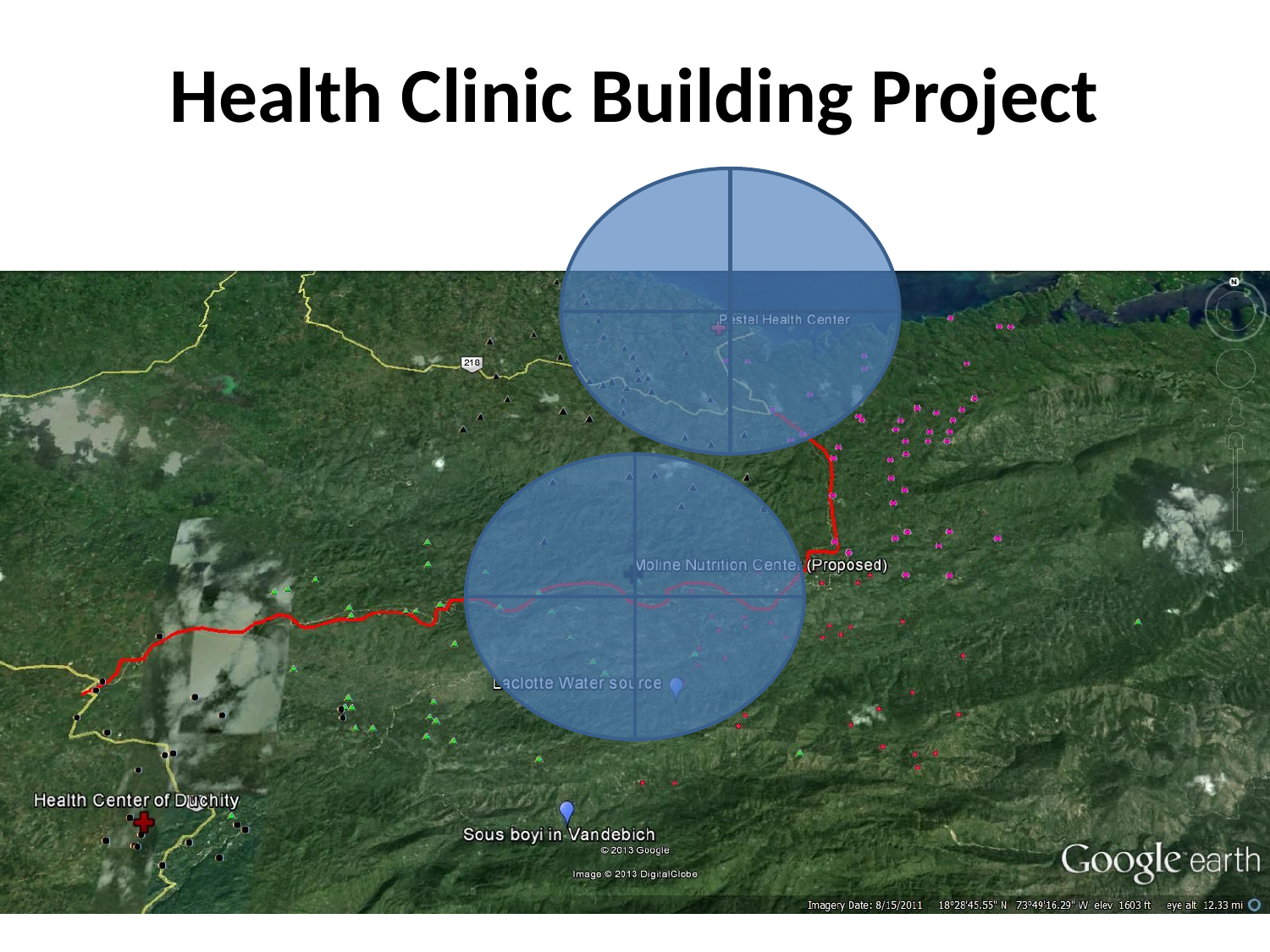

Health Clinic Building Project

## Slide 10
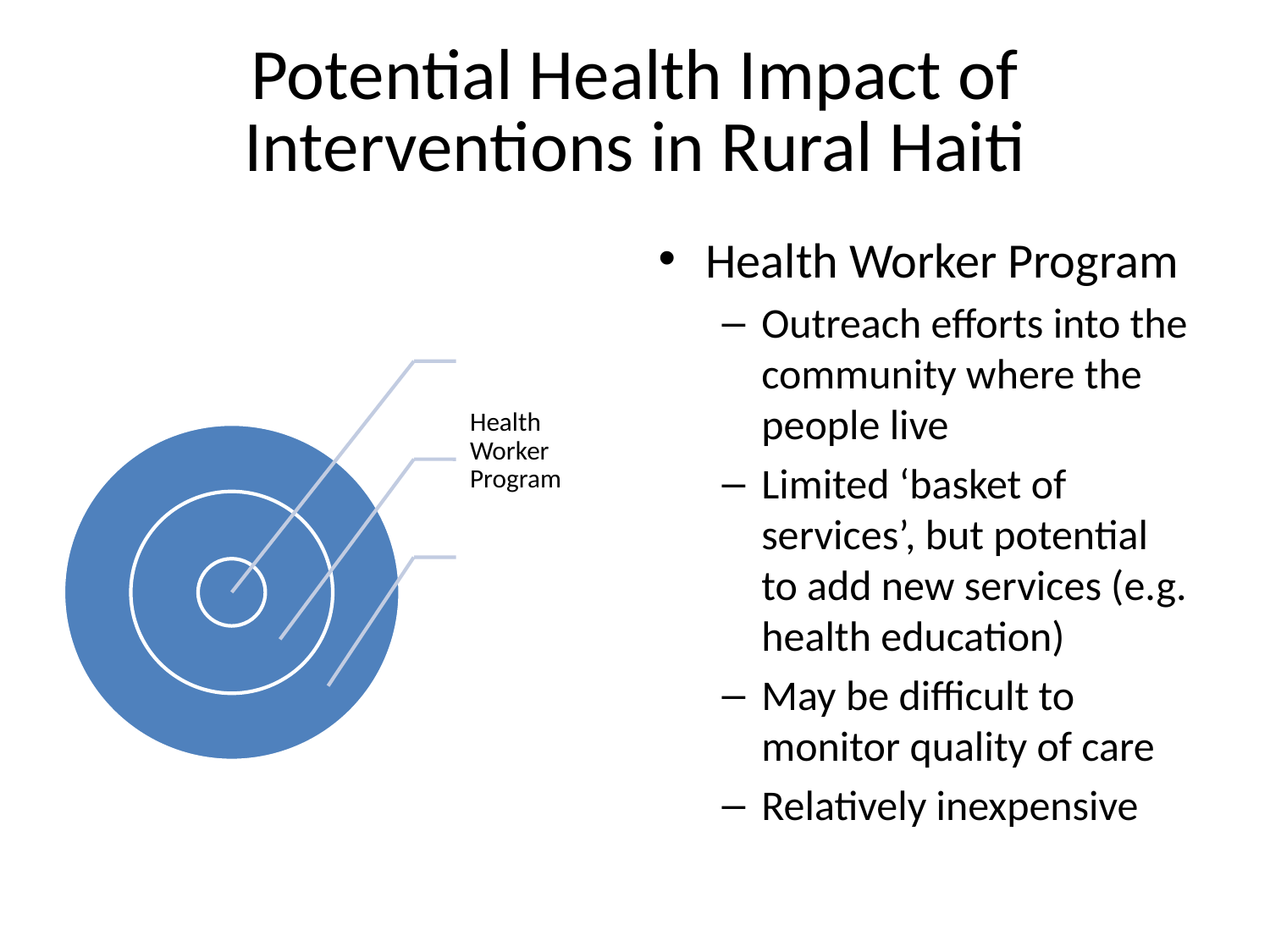

# Potential Health Impact of Interventions in Rural Haiti
Health Worker Program
Outreach efforts into the community where the people live
Limited ‘basket of services’, but potential to add new services (e.g. health education)
May be difficult to monitor quality of care
Relatively inexpensive

## Slide 11
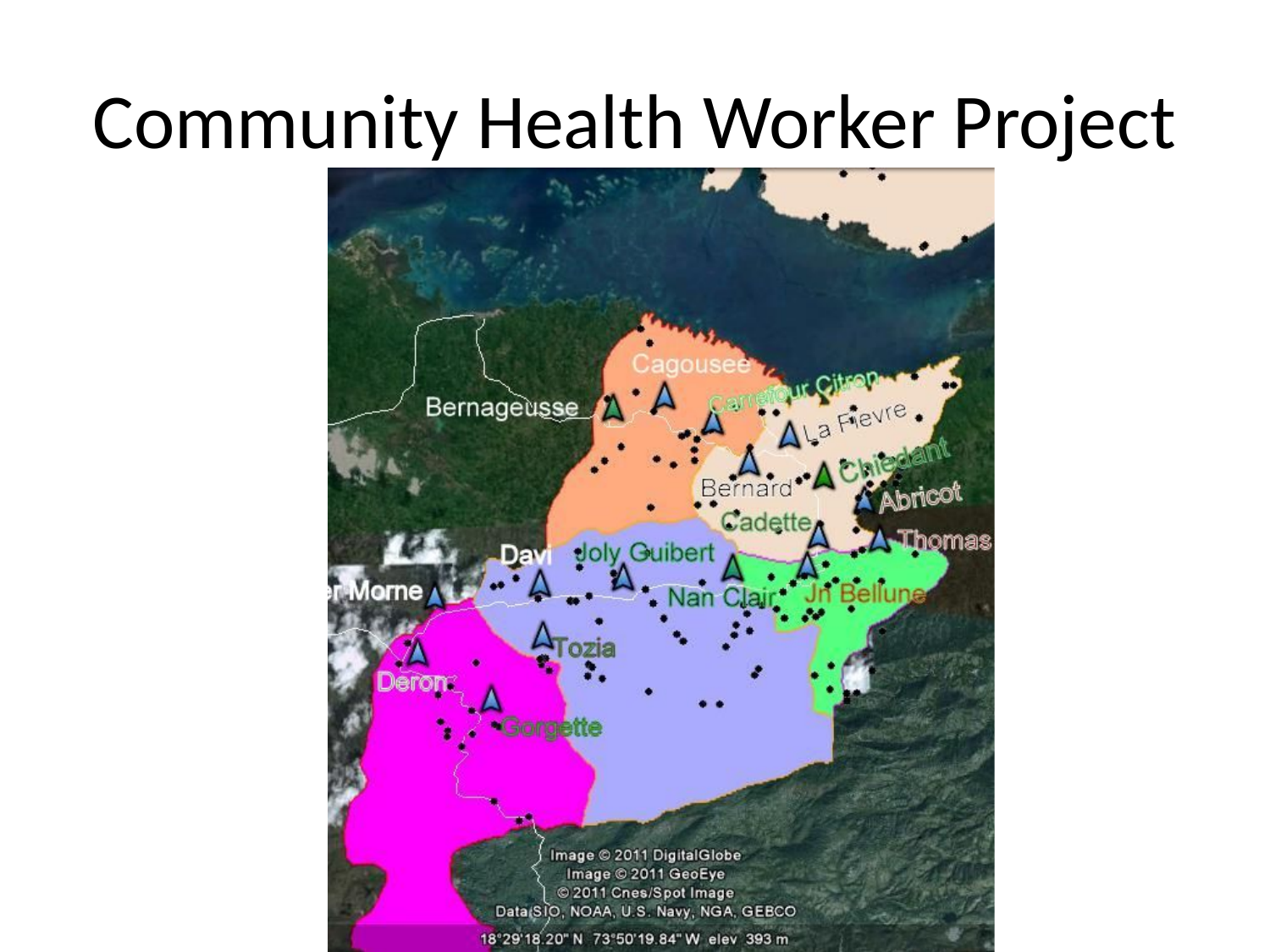

# Community Health Worker Project

## Slide 12
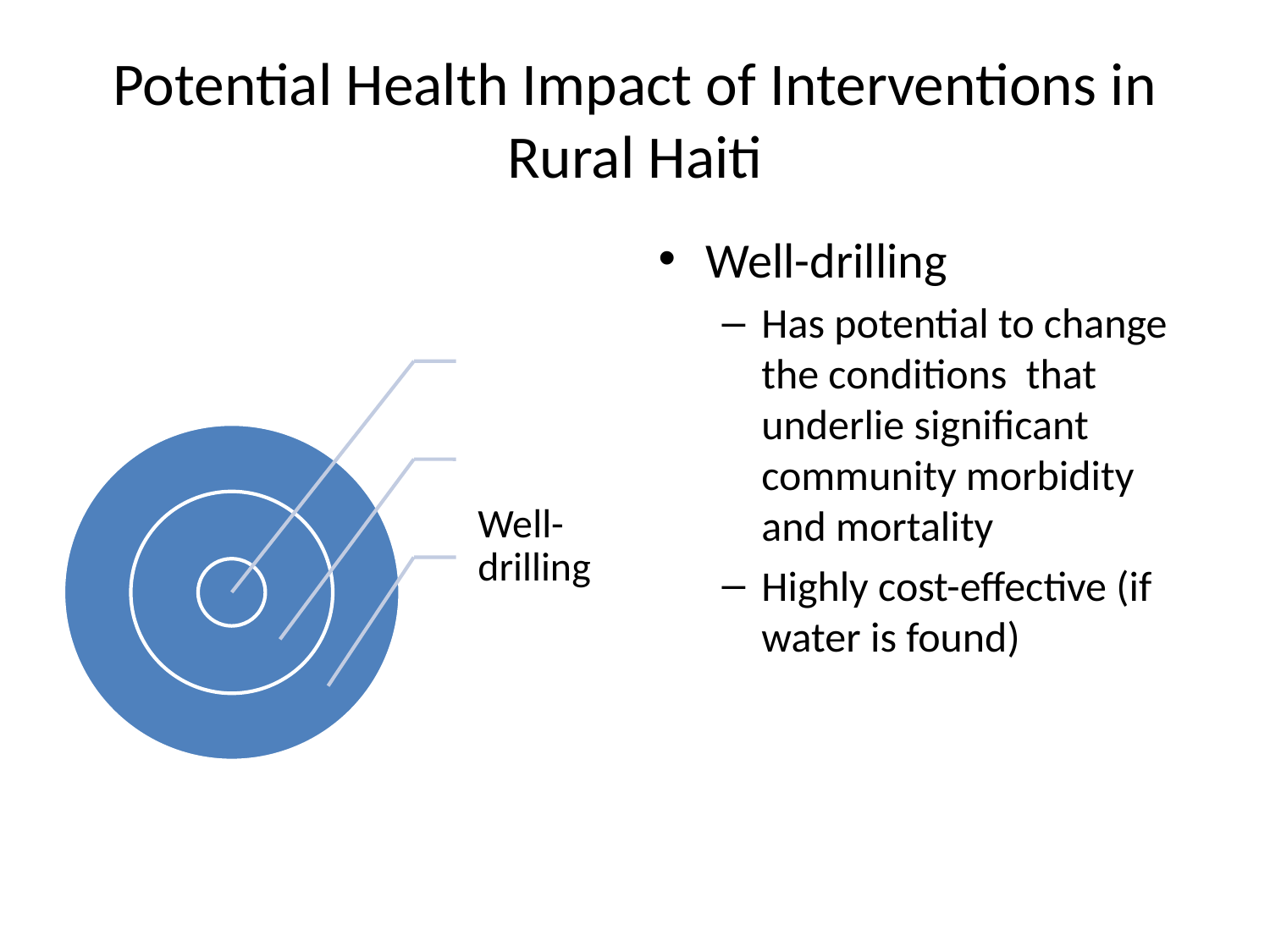

# Potential Health Impact of Interventions in Rural Haiti
Well-drilling
Has potential to change the conditions that underlie significant community morbidity and mortality
Highly cost-effective (if water is found)

## Slide 13
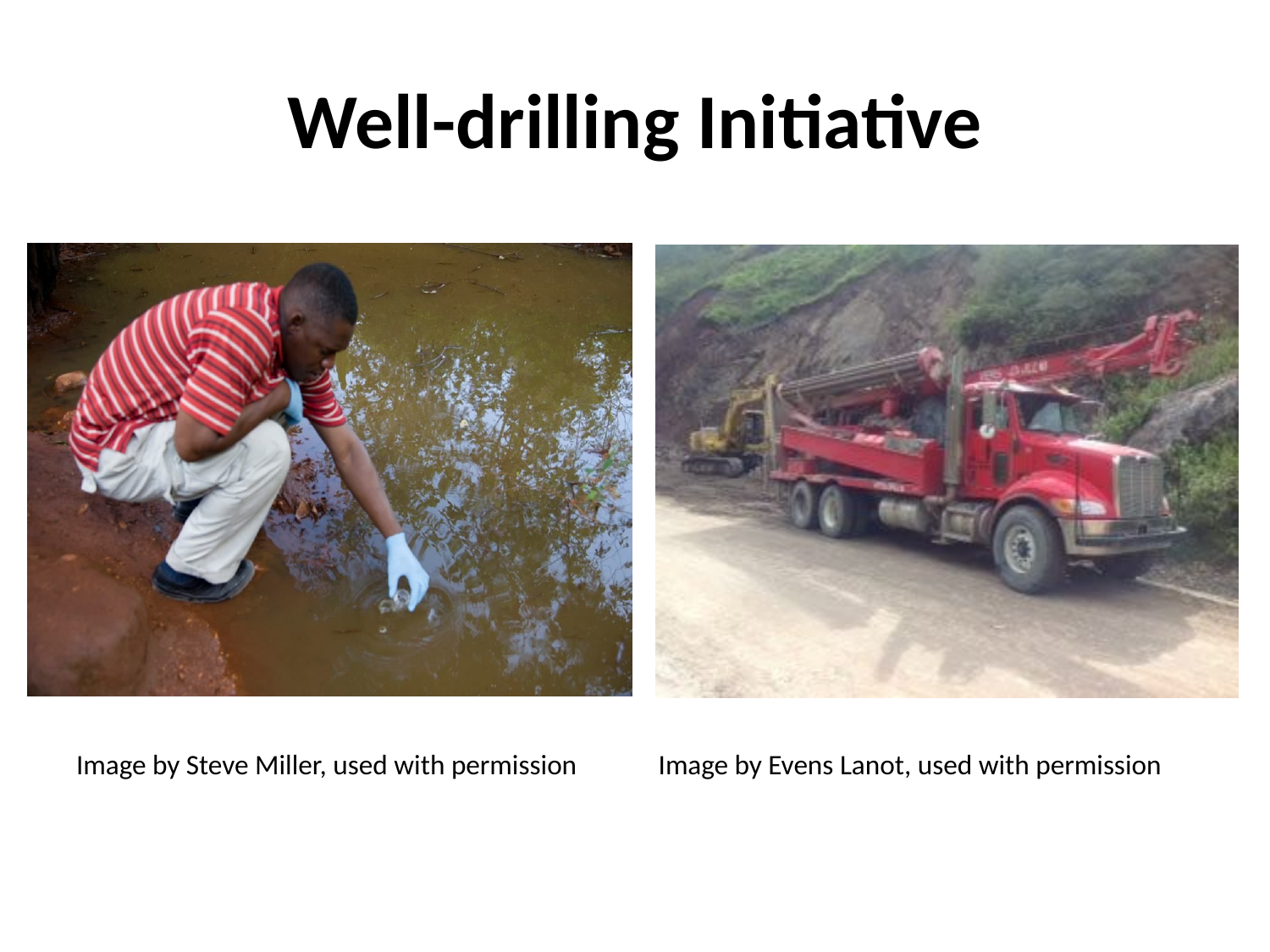

# Well-drilling Initiative
Image by Steve Miller, used with permission
Image by Evens Lanot, used with permission

## Slide 14
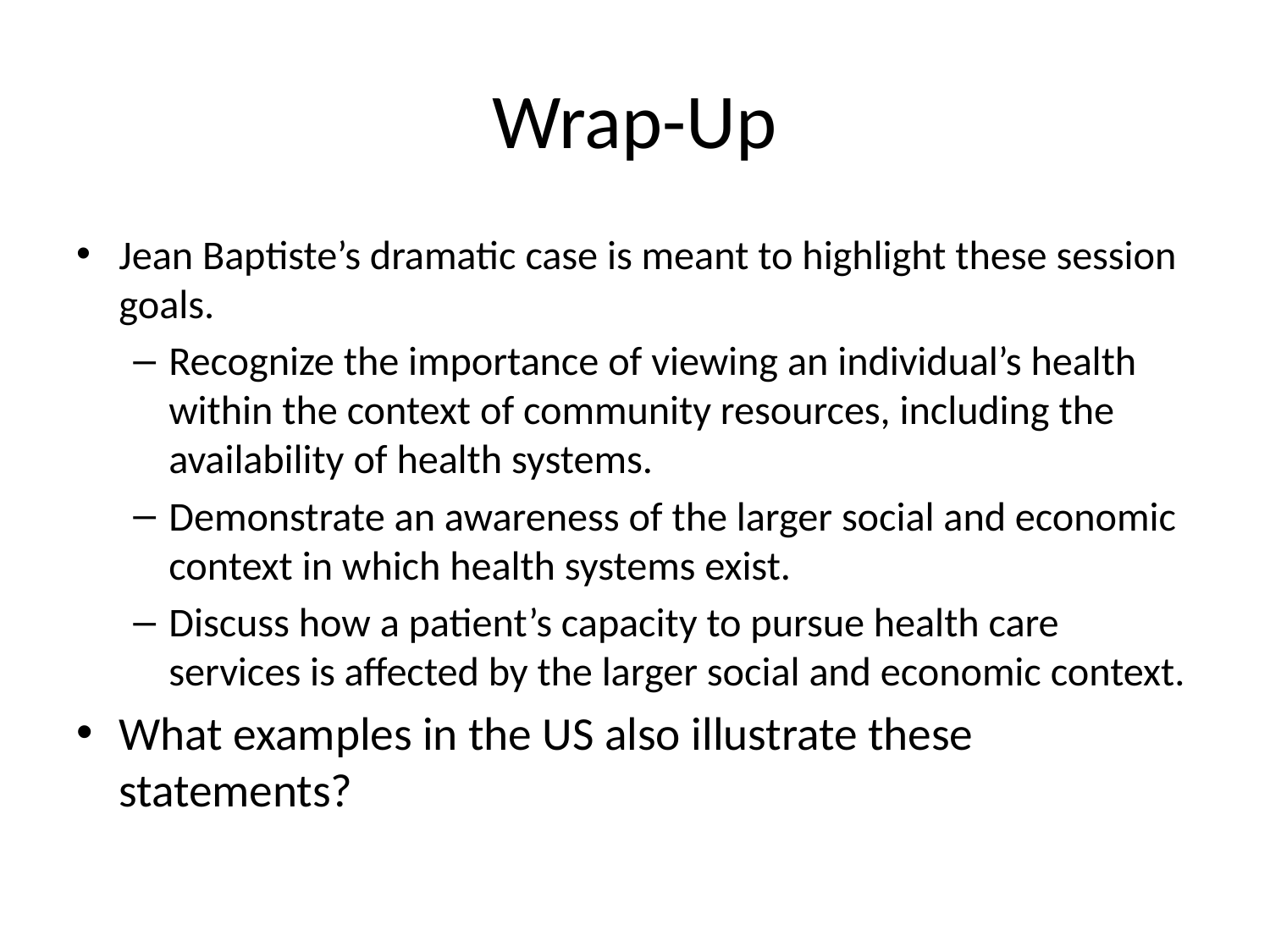

# Wrap-Up
Jean Baptiste’s dramatic case is meant to highlight these session goals.
Recognize the importance of viewing an individual’s health within the context of community resources, including the availability of health systems.
Demonstrate an awareness of the larger social and economic context in which health systems exist.
Discuss how a patient’s capacity to pursue health care services is affected by the larger social and economic context.
What examples in the US also illustrate these statements?

## Slide 15
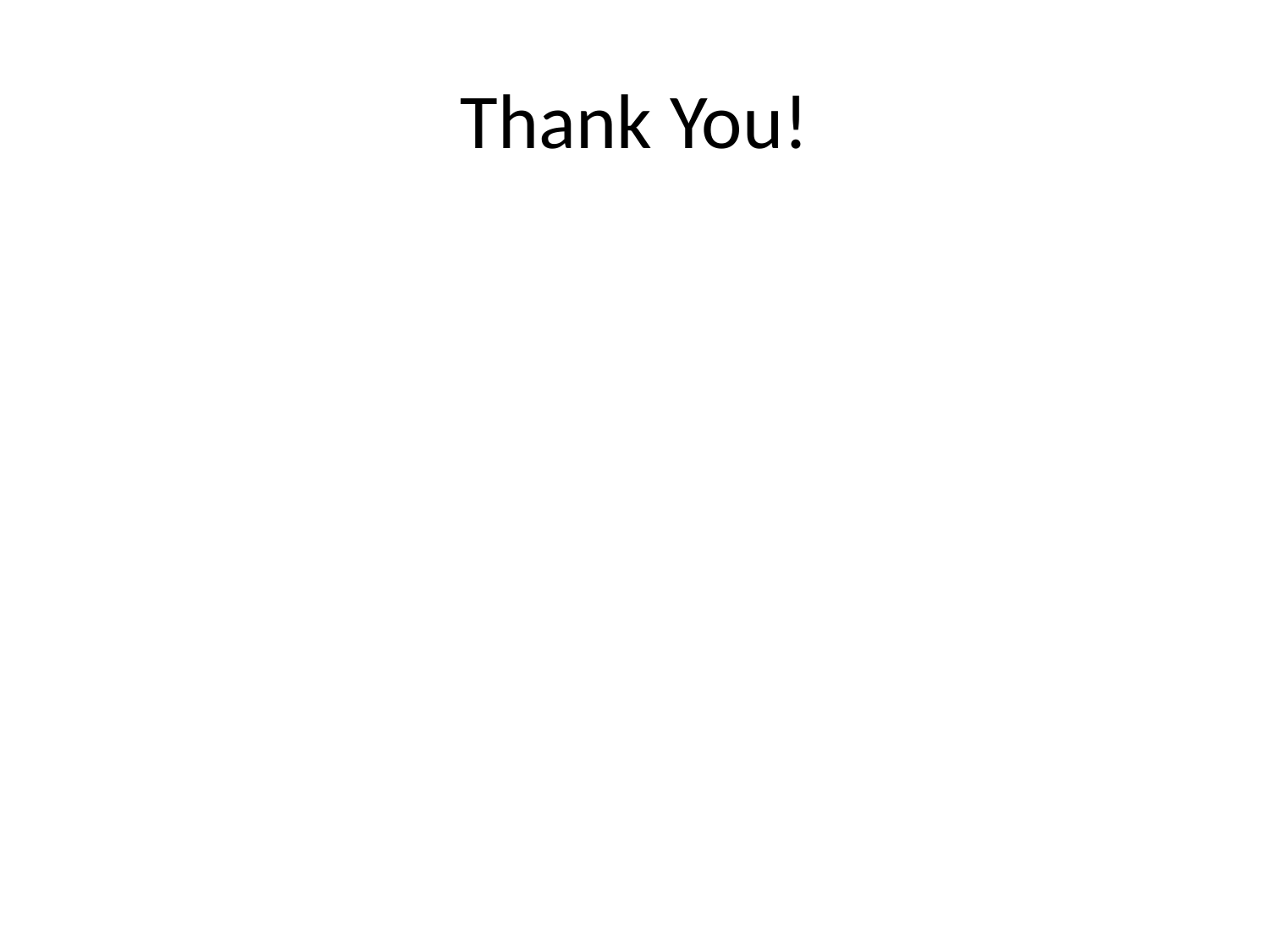

# Thank You!
