# Supplementary material for: Exploring Health Systems Within the Context of Social Determinants of Health: A Global Health Case Study
Source: MedEdPORTAL. 2016 Sep 23;12:10457. doi: 10.15766/mep_2374-8265.10457 (PMC6464409; doi:10.15766/mep_2374-8265.10457)
Supplement: Supplementary file 1 — A. Small-Group Case Study - Facilitator.docx B. Small-Group Case Study - Student.docx C. Large-Group Slides.pptx D. Large-Group Facilitator Guide.docx E. Additional Case Details.pdf [file mep-12-10457-s001.zip › D. Large-Group Facilitator Guide.docx]

# Large-Group Facilitator Guide

**Large Group Activity: 1 hour**

Progression of Sessions:

1. The small groups will start by discussing a patient who has a health issue, then move into discussion about the social and economic context around the patient’s health issues. The small group session will end with some broader questions in preparation for the large group discussion on community health. This case of a Haitian boy with rheumatic heart disease highlights the importance of social and economic factors as major contributors to the disease. Arriving at the correct diagnosis at the end of page 1 is not as important as having the students struggle with the questions of WHY this preventable condition happened in the first place.
2. The large group session will provide learning and discussion of broader themes of how an individual’s health, and how health systems in general, are affected by the social, economic, and geographic factors. The large group session will focus on the importance of viewing an individual’s health within the context of community and the availability of health systems.

**Preparation:**

1. Print off packets (Appendix E) for each small group. One set of printouts per group. Distribute packets in small group setting. Small groups will open packets and review the information during the large group session.
2. Print off voting cards (A, B, C, D) or use an audience response system.
3. Review Large Session Powerpoint (Appendix C) slides.


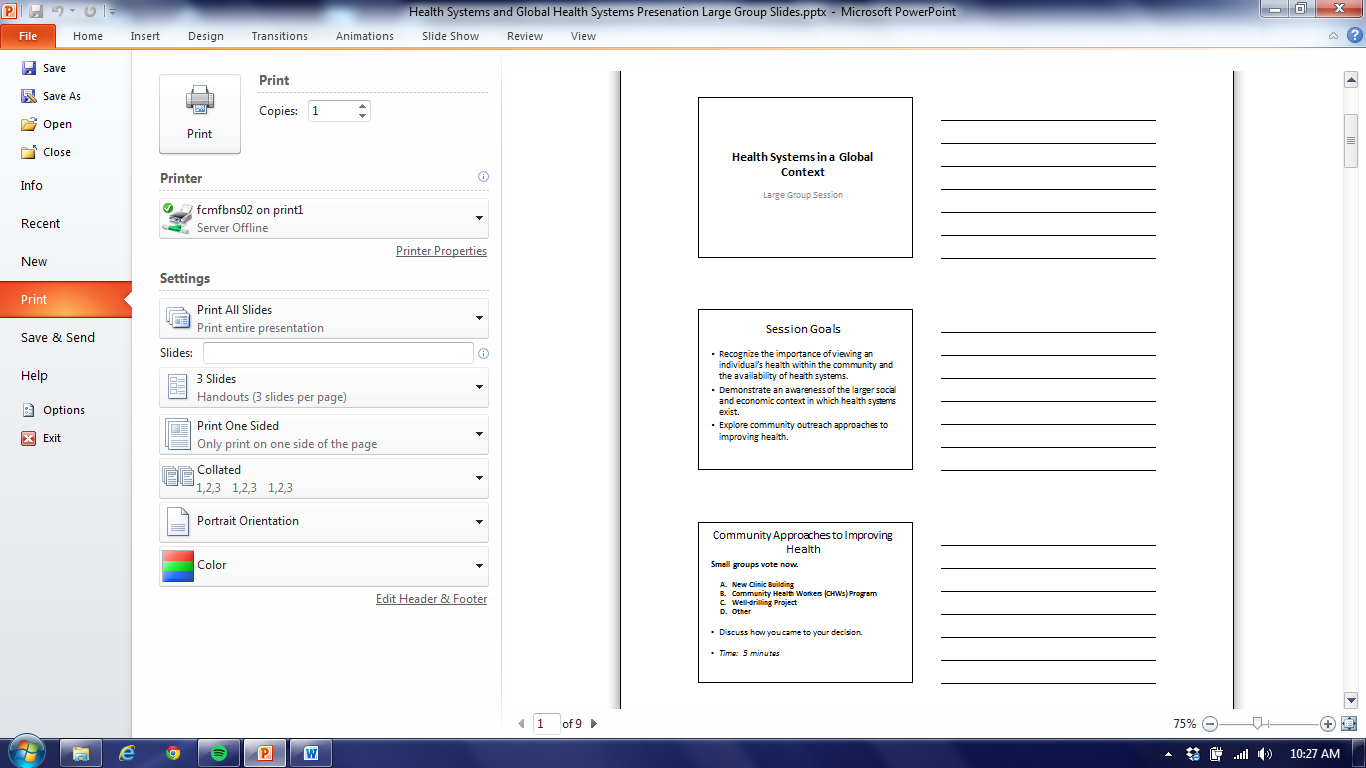


Review the Learning Objectives.

The case study highlighted a boy who did not have access to a basic health system. His situation highlights how social, economic and environmental factors affect the availability of health systems, even in their most basic forms.

When small groups are ready to begin, welcome them to the large group session. This session will continue the discussion of health systems in a global context.

This session intends for the small groups to learn from one another.

Each group Votes.

What did your groups decide on? How did your group vote? What were some of the reasons for voting as you did?

What are some of the questions you raised in your group?


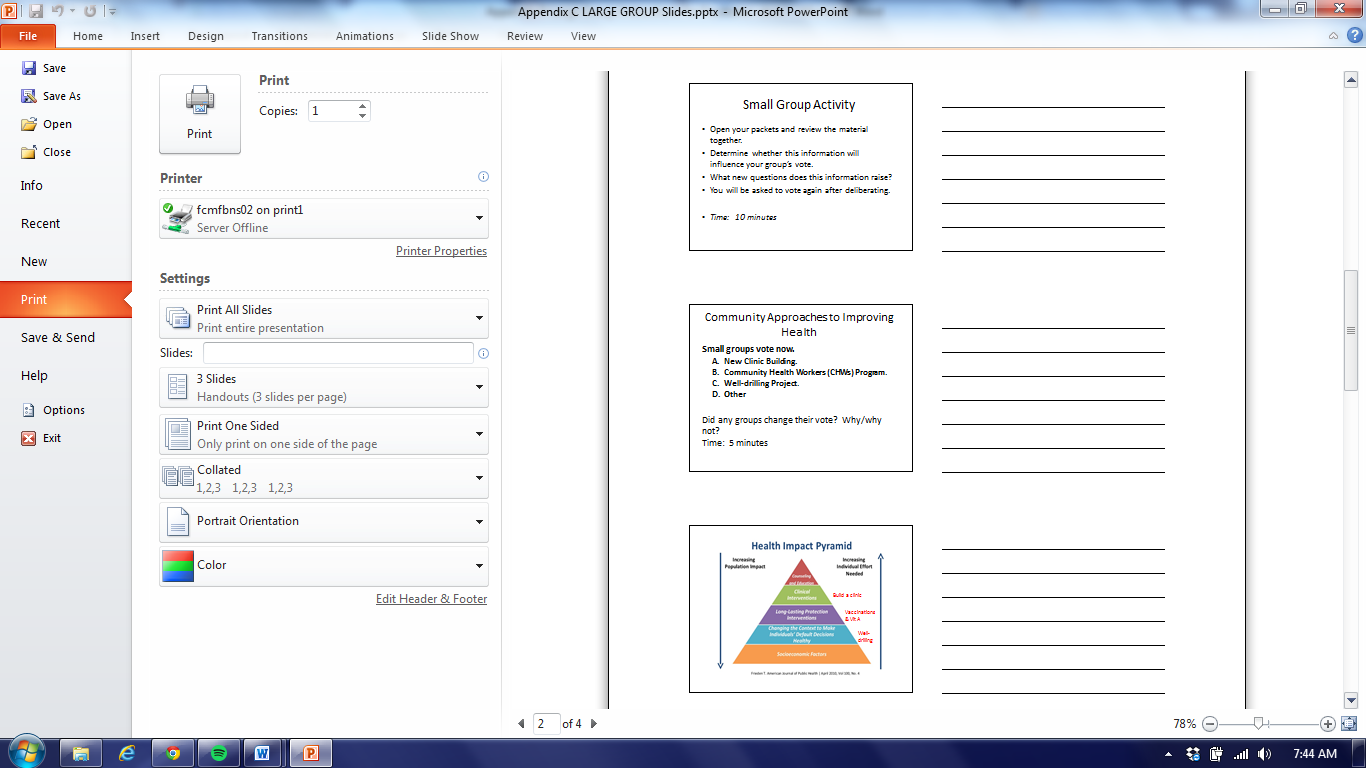


Each group Votes again

Did any group change the vote?

What new questions did the information raise?

Each group opens their packets. The packets contain the same information that describes the social-ecological context in rural Haiti.

Instructions to small group: Consider what details in this packet might affect your decision about which health intervention might be feasible. There is no “Right” answer. This session is interested in helping students think through the context in which health systems exist (or are lacking).


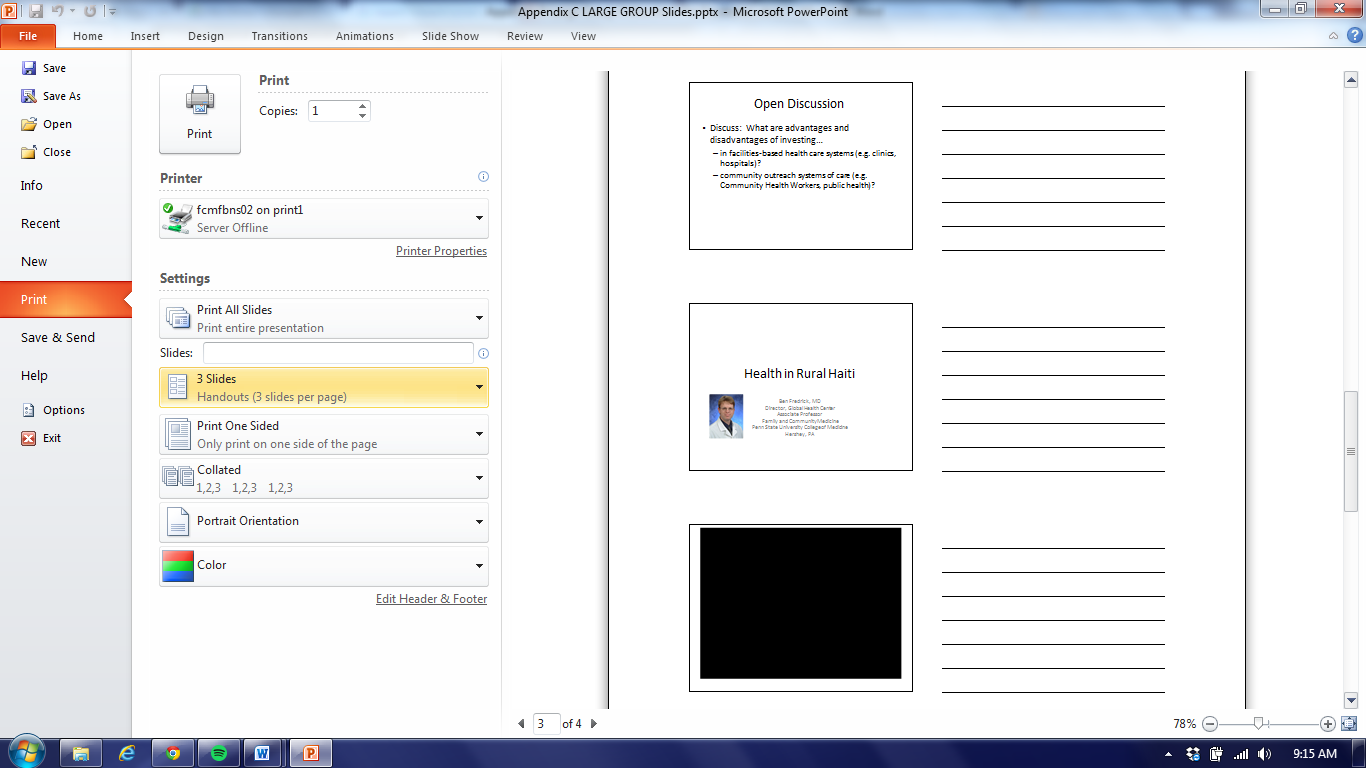


Lead an open discussion on this question.

This slide demonstrates the expected “catchment” area of the existing health center (along the coast) and the proposed new clinic site. Large areas of the population remain unserved.

While it may be natural for physicians and health professionals to consider a health clinic, typically this is a costly undertaking requires supply chains to maintain stock of medicines and supplies. In rural settings like Haiti it is common for people to travel great distances to reach a clinic, and often they present very late in the course of the disease. In short, those most in need remain among the least benefited by a health clinic. Health clinics continue to serve important functions within the community’s health system, but in the context of social and economic deprivation such as this, attention should first be paid to the public health basics.

The next set of slides graphically represents the potential impact that each intervention has on the health of a community. While the health clinic is at the center, this truly represents a limitation of reach out into the community.

Changing the environmental conditions such as well-drilling in a region of great water scarcity has potential save thousands of lives for years to come. Poor water access and poor quality water underlie a tremendous degree of mortality, especially among children, in places like rural Haiti. Diarrhea is the #2 cause of child mortality globally, and issues of water are at the heart of that problem.

The map shows all the villages that the Health Workers reached and documented with GPS coordinates. Through the Health Worker Program ~10,000 children were reached twice a year with Vitamin A and deworming.

Community Health Workers as a concept were developed in impoverished communities like rural Haiti in order to reach the most in need with basic health care services like Vitamin A and vaccinations. Community Health Workers are now being adopted in different communities within the United States because of the value they bring to the health system and the community.

Photos from the actual area.
